# Supplementary material for: The effects of death and post-mortem cold ischemia on human tissue transcriptomes
Source: Nat Commun. 2018 Feb 13;9:490. doi: 10.1038/s41467-017-02772-x (PMC5811508; doi:10.1038/s41467-017-02772-x)
Supplement: Supplementary file 1 — Supplementary Information [file 41467_2017_2772_MOESM1_ESM.pdf]

Figures

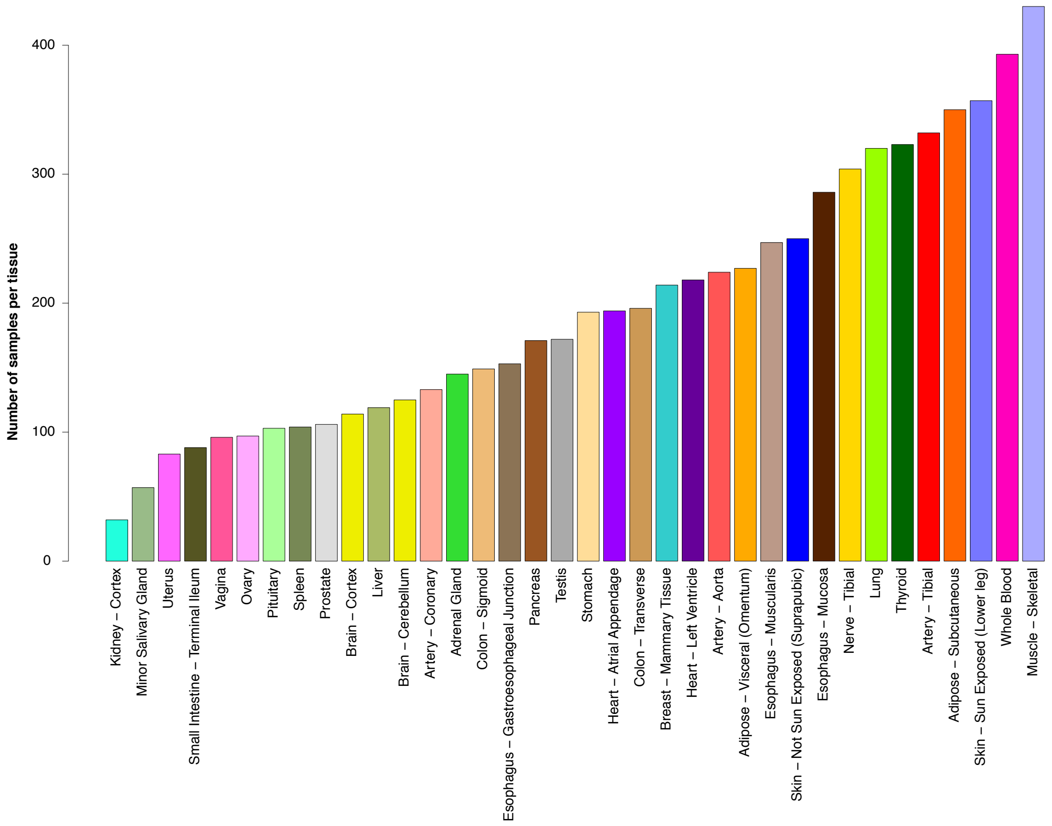

Supplementary Figure 1: Number of analyzed samples across different tissues. 36 tissues with more than 20 samples (mean sample size: 193, min: 32, max: 430) were considered for the current study (average 13 samples/donor).

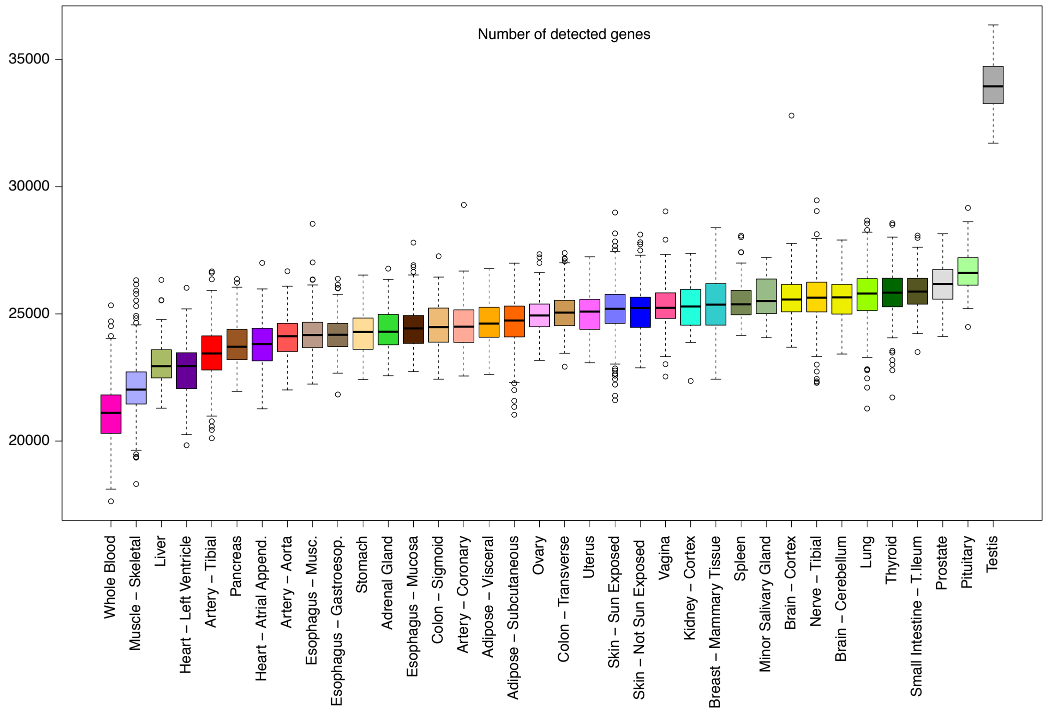

Supplementary Figure 2: Number of genes with minimal expression detected across different tissues. Distribution of the number of genes detected with at least five mapped reads, including all the biotypes in Gencode v19 annotation (56321 annotated genes).

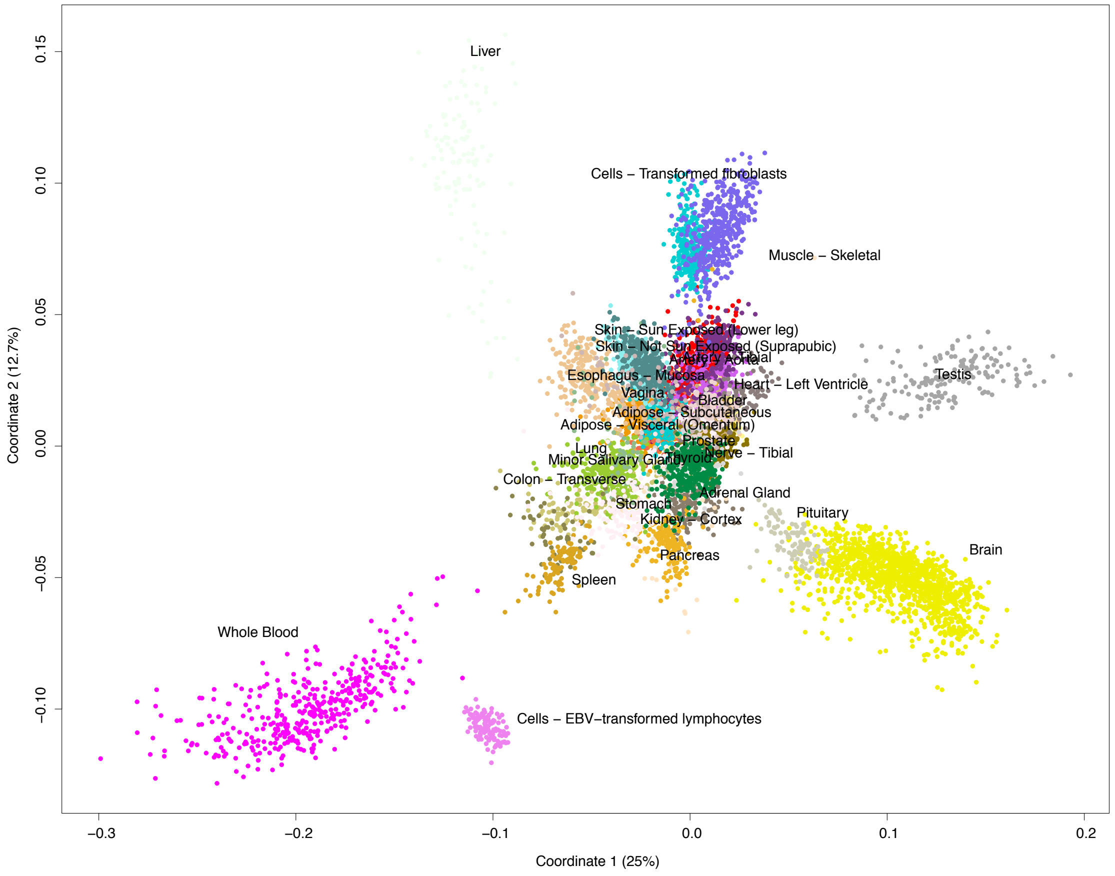

Supplementary Figure 3: Multi-Dimensional Scaling of 8555 GTEX samples. Multidimensional scaling based on gene expression profiles of all annotated genes shows that the main driver of sample similarity and variability is tissue of origin.

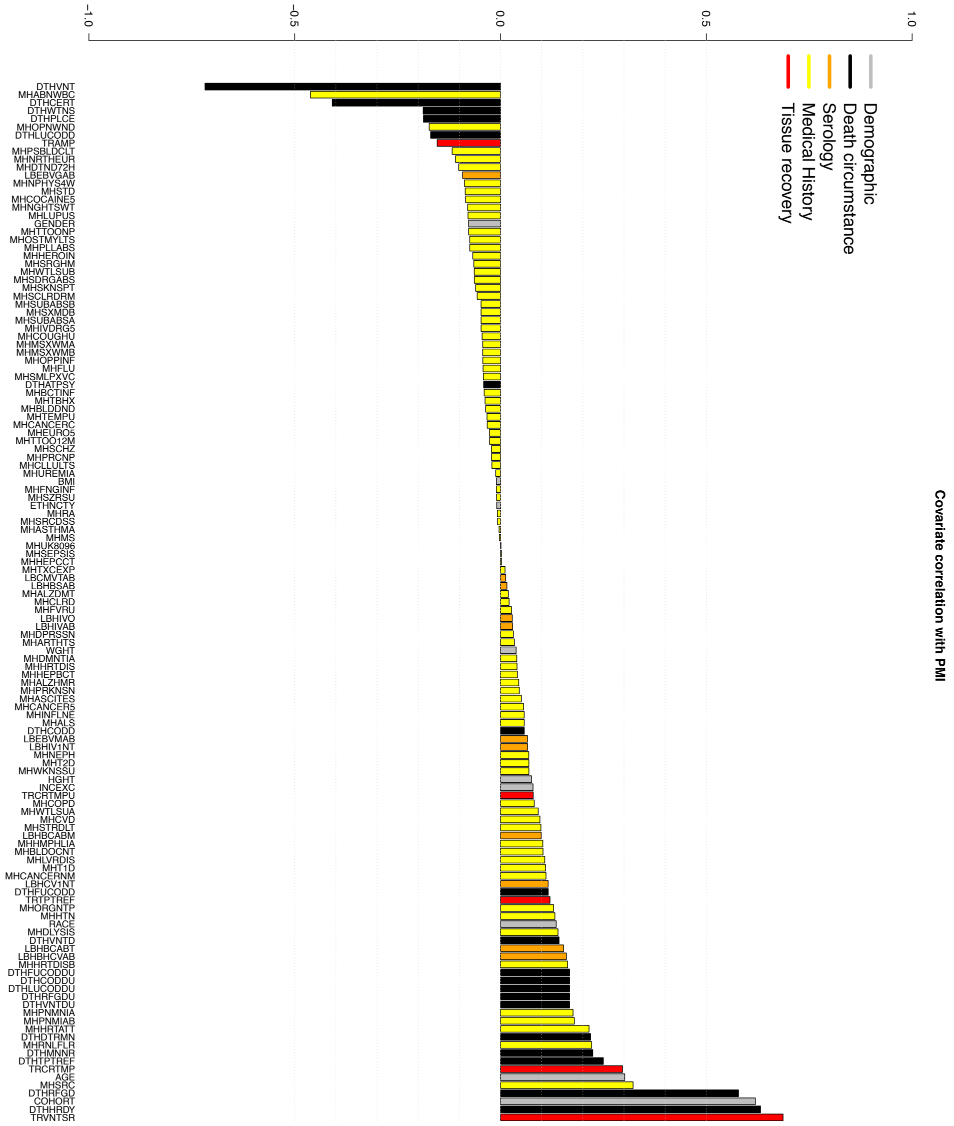

Supplementary Figure 4: Correlation between PMI and the different annotated covariates. Covariates are colored by respective five different categories. Correlation values and description of the covariates is provided in Supplementary Table 3.

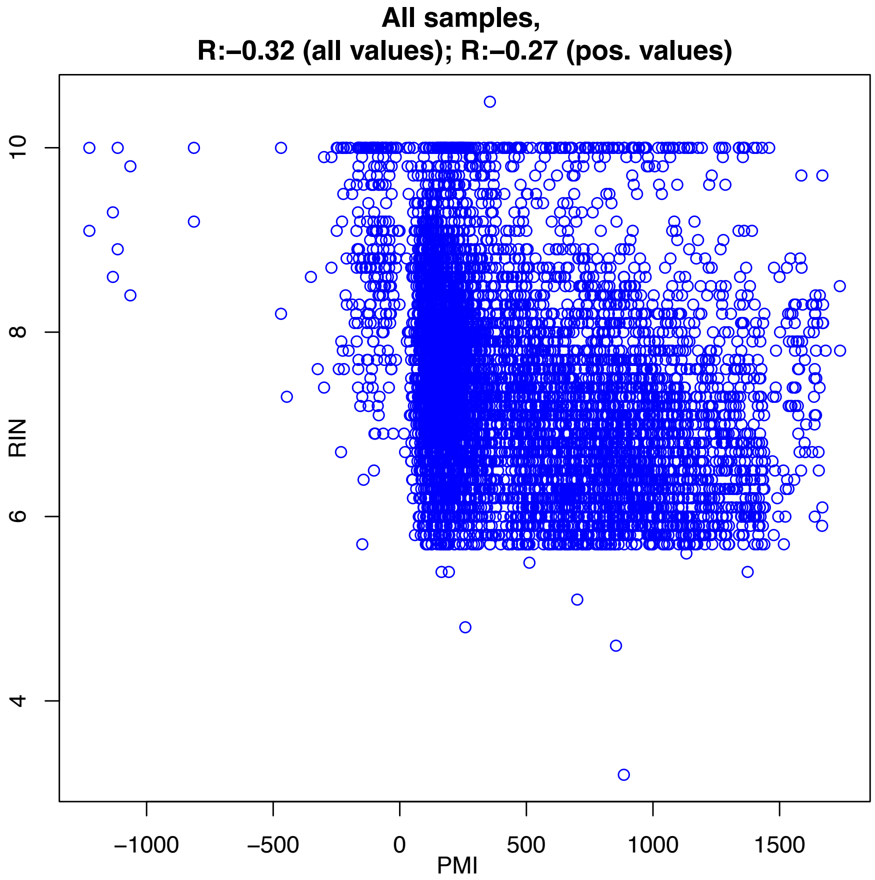

Supplementary Figure 5: Relation between PMI and RIN values for all the samples. No notable association is observed when considering all samples, where Pearson correlation r=-0.32 for all samples and r=-0.27 when excluding Blood pre-mortem samples.

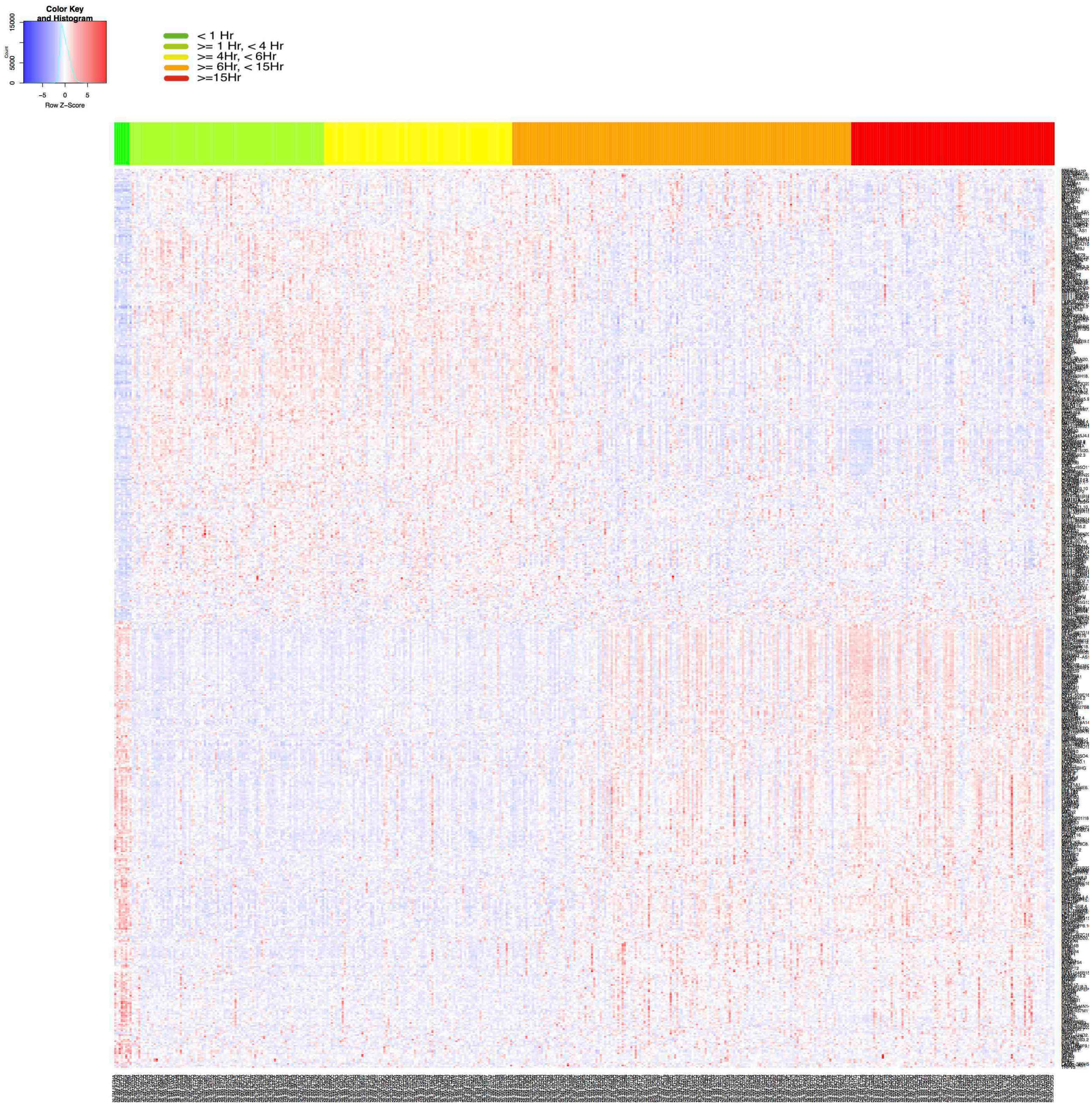

Supplementary Figure 6: Heatmap of normalized gene expression for genes with temporal differential expression in Muscle. Rows represent genes with significant change and the top bar indicates by the color code the PMI interval for the samples.

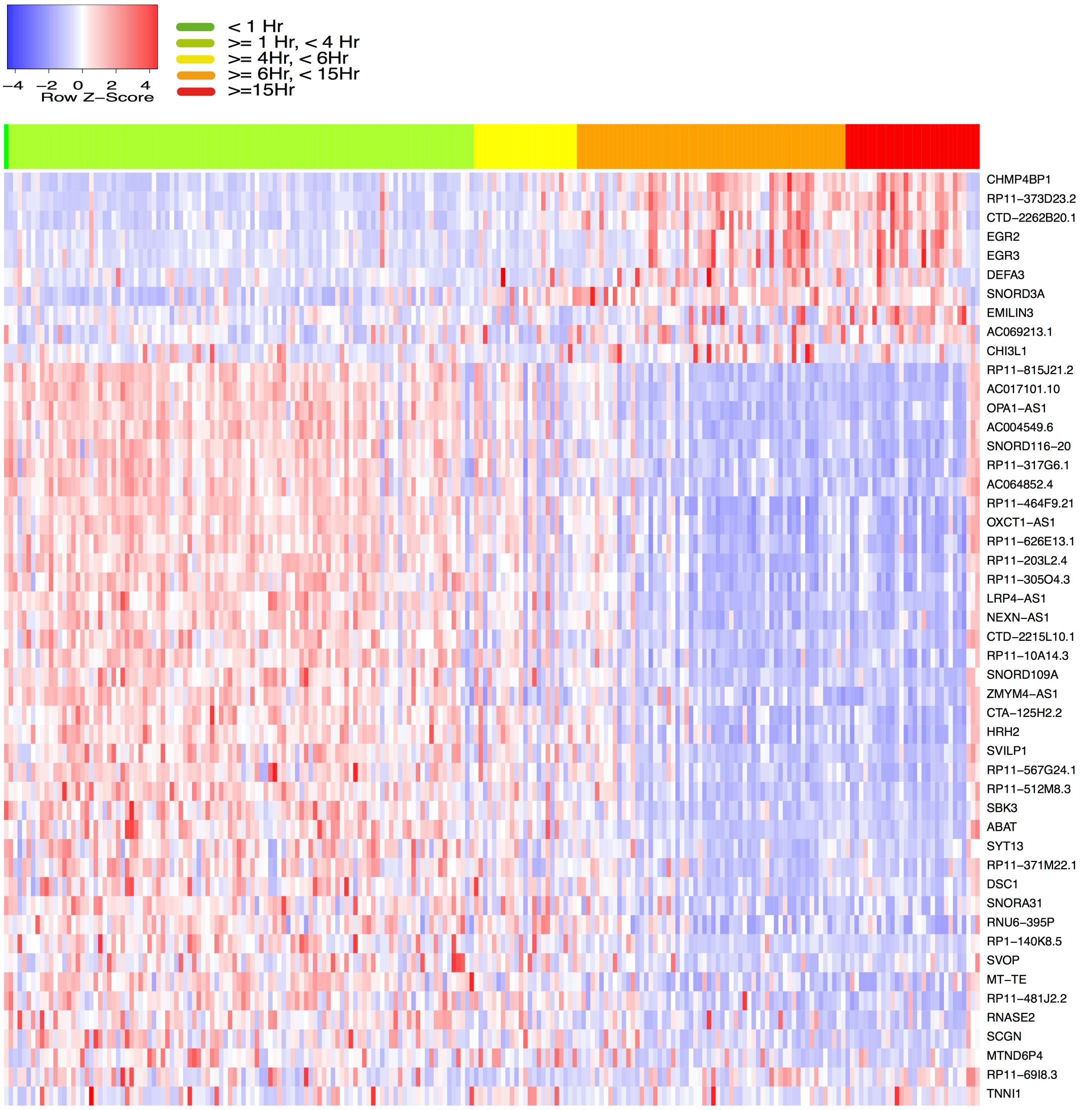

Supplementary Figure 7: Heatmap of normalized gene expression for genes with temporal differential expression in Heart - left ventricle. Rows represent genes with significant change and the top bar indicates by the color code the PMI interval for the samples. We detect a steady-state change with genes increasing their expression at later (6 hours) PMI time points and another group of genes decrease their expression at later (6 hours) time points.

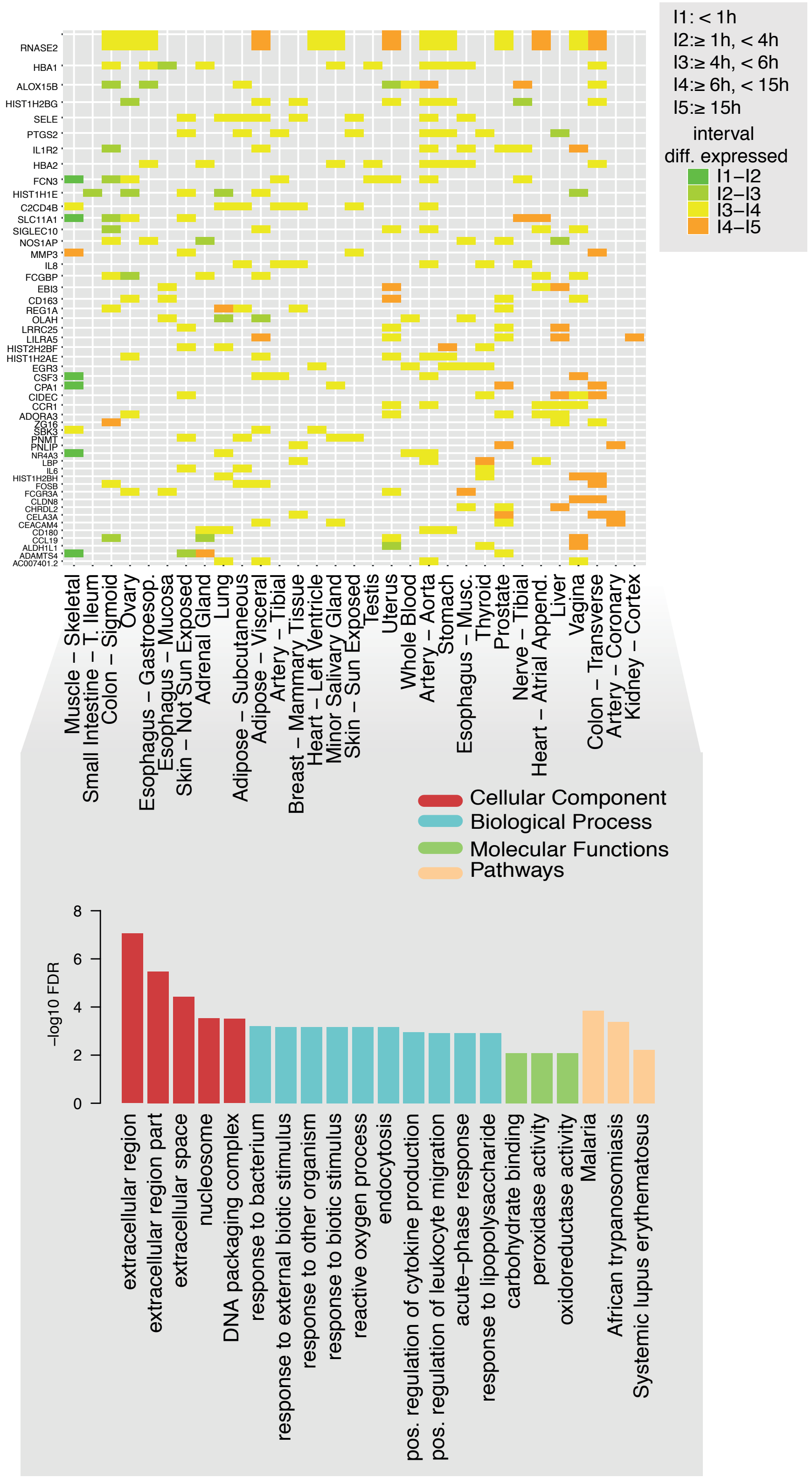

Supplementary Figure 8: Analysis of genes with non-linear temporal differential expression recurrently across tissues. (Top) Matrix with the genes with significant change and the interval where the change occurs. Only top recurrent protein coding genes are shown. Rows correspond to genes recurrent in three or more tissues. Genes are sorted by their frequency and tissues are sorted by the average value of the interval in which differential expression occurs. Changes at later PMI are more frequent. (Bottom) Gene Ontology and Pathway analysis of these genes.

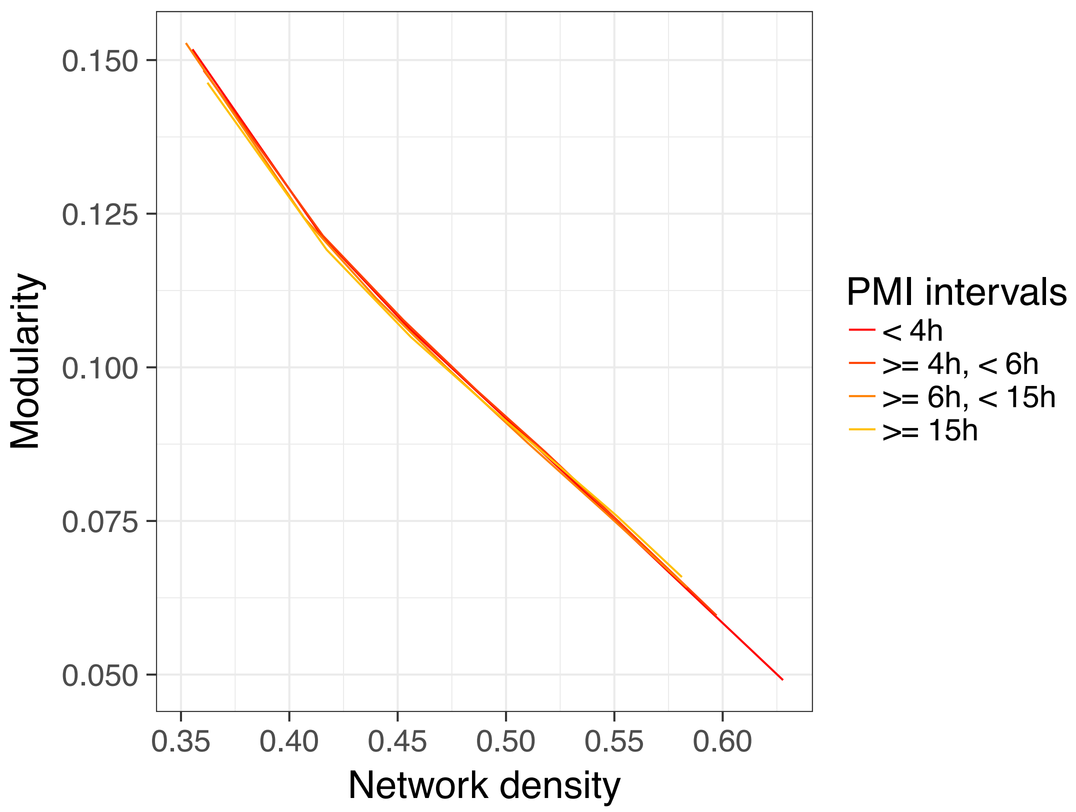

Supplementary Figure 9: Clustering Modularity. Distribution of network density with relation to modularity in the four combinations of samples.

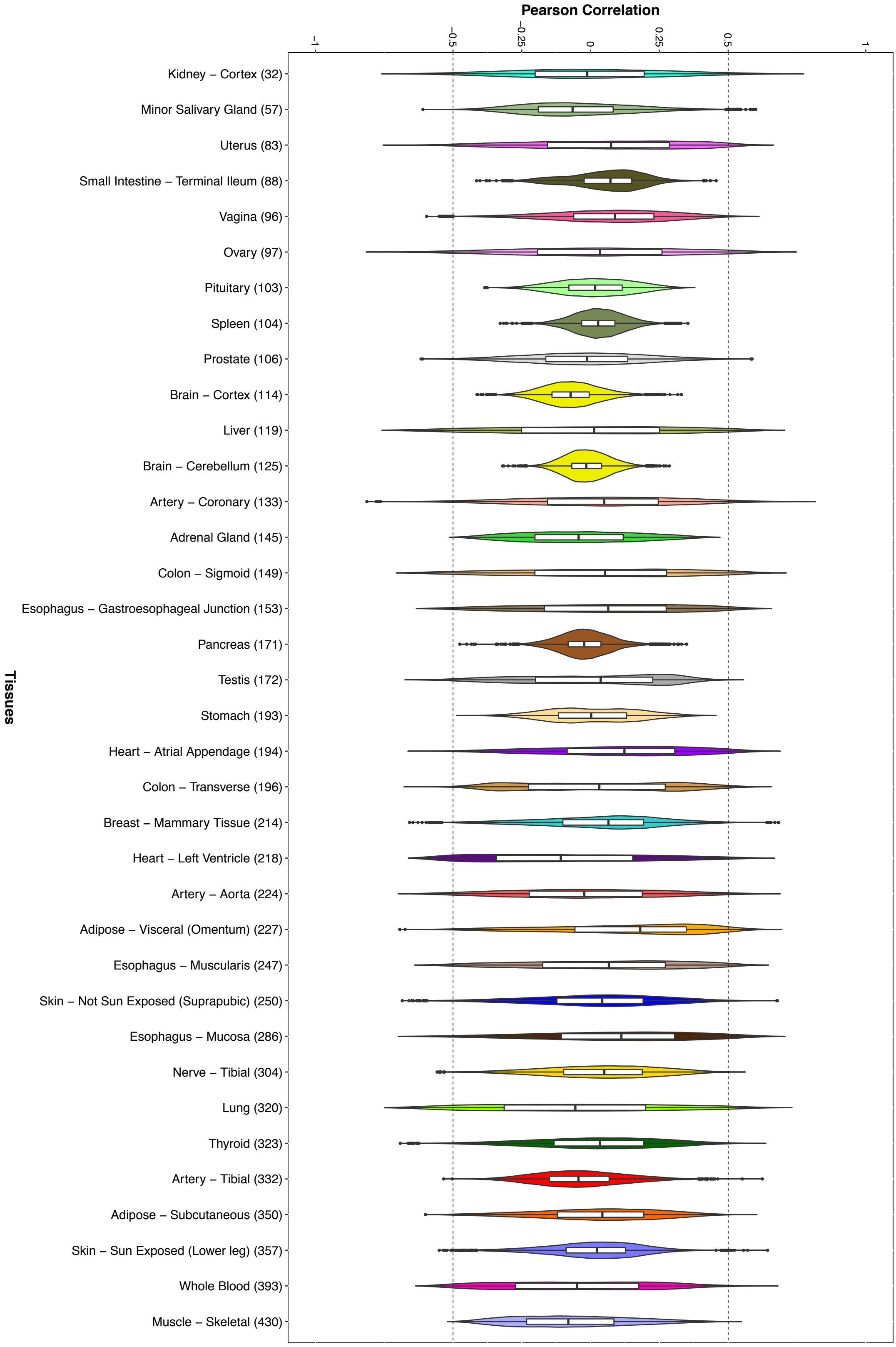

Supplementary Figure 10: Distribution of correlation of gene expression and PMI without considering covariates. Tissues are sorted by sample size. Dashed line represents a 0.5 correlation threshold.

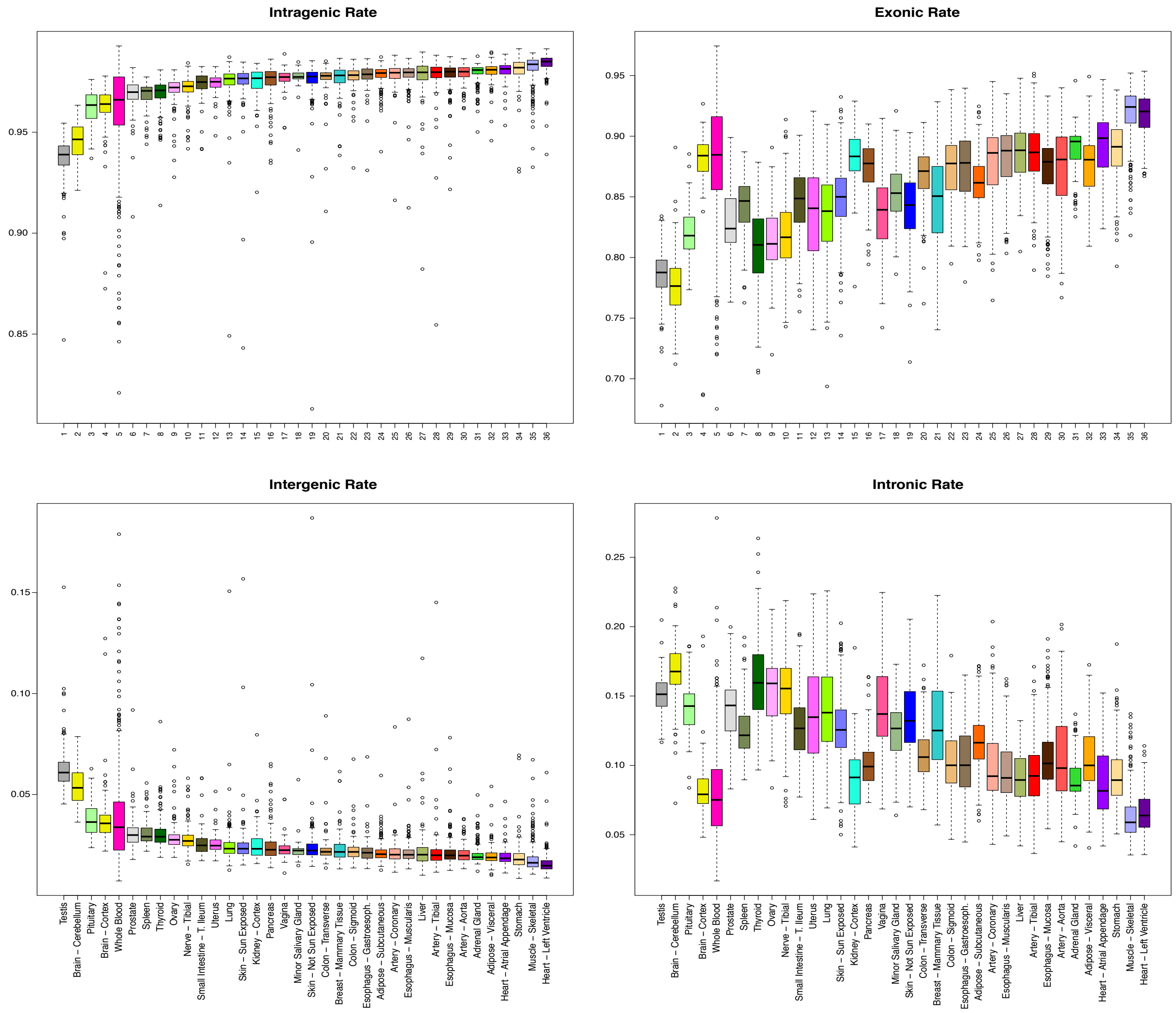

Supplementary Figure 11: Proportion of read coverage of intragenic (exonic and intronic) and intergenic features across different tissues. Intragenic and intergenic proportions sum to 1.

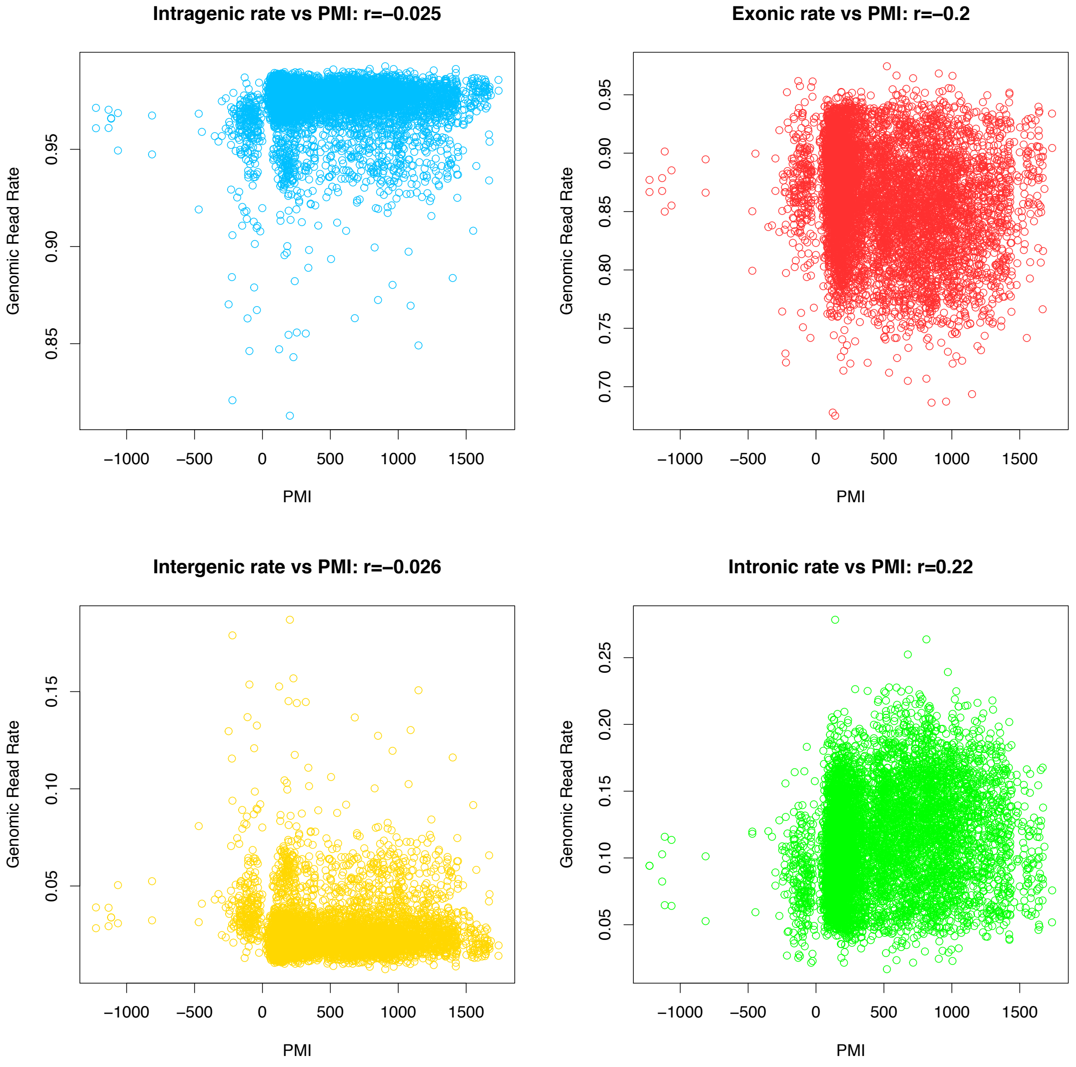

Supplementary Figure 12: Genomic Read Rate in intergenic and intragenic regions with relation to PMI. The percentage of intragenic and intergenic read coverage for all the samples and the respective PMI values show low correlation.

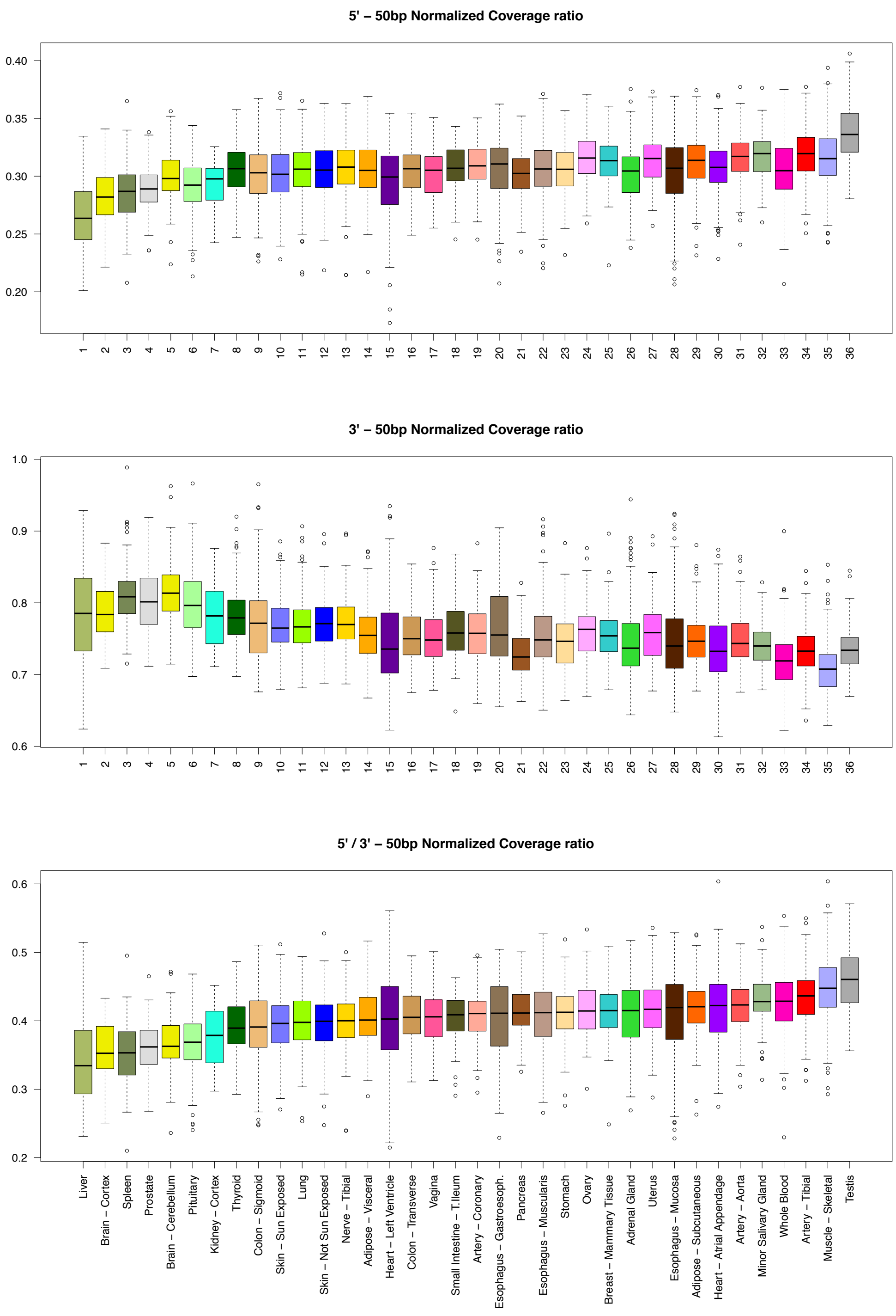

Supplementary Figure 13: Normalized read coverage along the 5' and 3' regions and respective ratio. Upper and middle plot: correspond respectively to the 5' and 3' 50 bp-based normalization; this value is the ratio between the coverage at the 5' or the 3'end and the average coverage of the full transcript, averaged over all transcripts; bottom plot, respective ratio between the values in the 5' and 3' normalized read coverage.

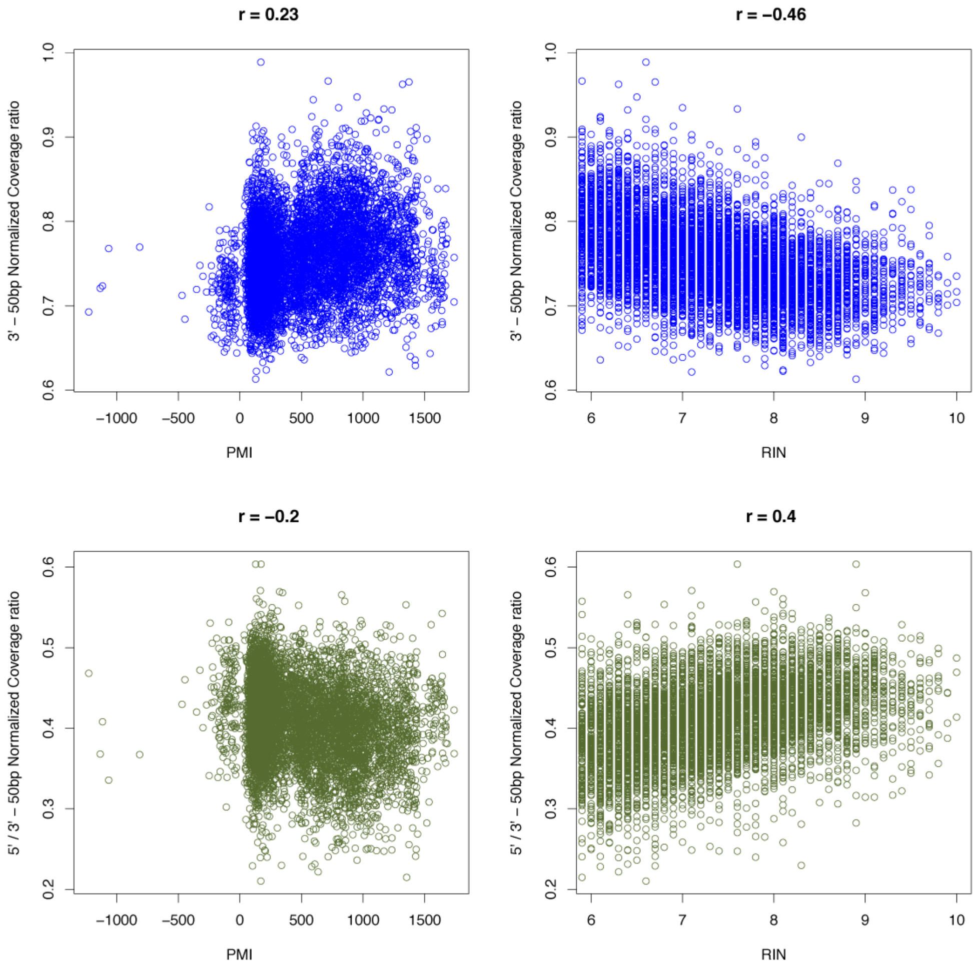

Supplementary Figure 14: Association between mapping bias and PMI and mapping bias and RIN values for all samples. Upper plots: correlation between 3' 50 bp-based normalization and PMI and RIN. Bottom plots: correlation between 5'/3' 50 bp-based normalization ratio and PMI and RIN.

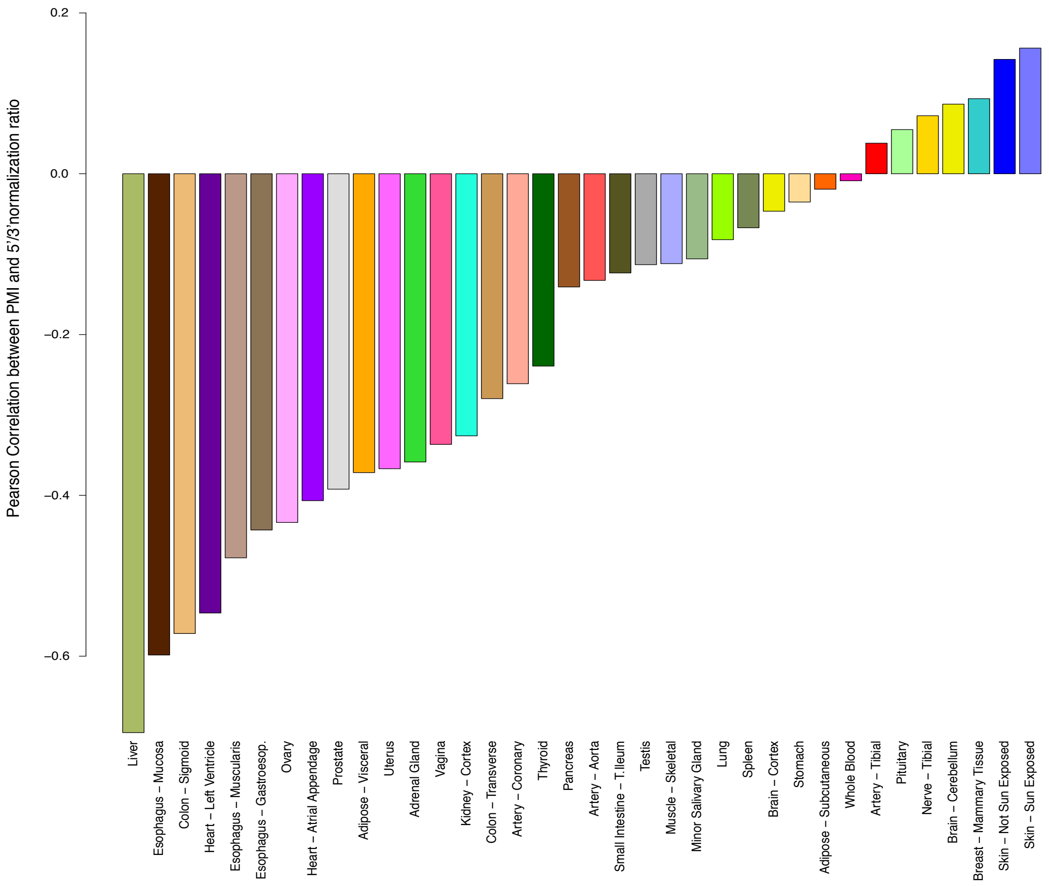

Supplementary Figure 15: Association between PMI and mapping bias by tissue. Association between 5'/3' 50 bp-based normalization ratio and PMI show distinct behavior for the different tissues.

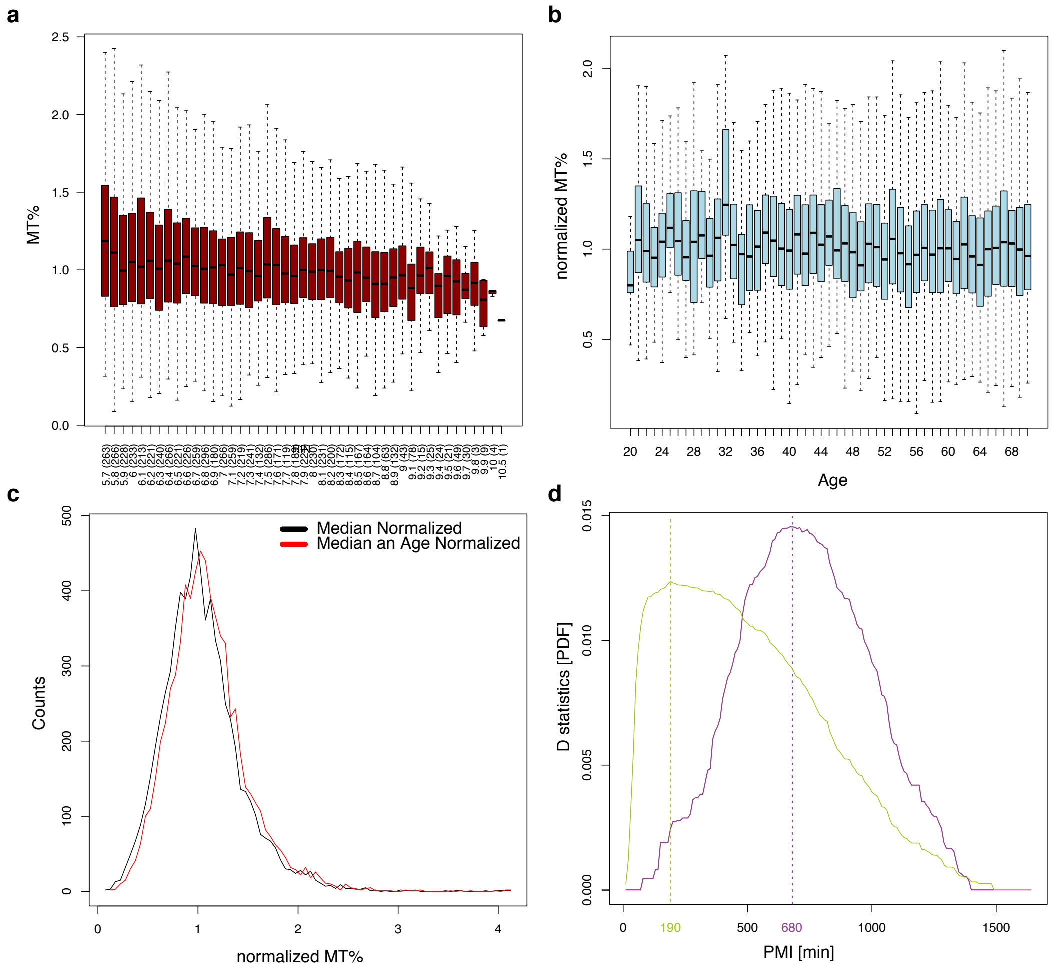

Supplementary Figure 16: Concentration of mitochondrial RNA. a) Distribution of proportion of mitochondrial reads with relation to RIN values. Numbers in the x-axis indicate the number of observations for the respective RIN value. Relative concentration of mitochondrial gene copies is relatively equivalent across the different RIN bins. b) Boxplot of median normalized proportion of mitochondrial reads. No significant Pearson correlation was found between donor age and normalized mitochondrial proportion (p-value = 0.211). c) Comparison of normalized mitochondrial proportion values after age correction according to a least squares linear model fit. d) The D statistics (MWW test) for the differences in mitochondrial RNA concentrations between short and late times of PMI reaches a maximum at 190min of PMI considering all post-mortem samples (dashed green line), and a maximum at 680min for the postmortem samples (excluding organ donor samples) (dashed purple line).

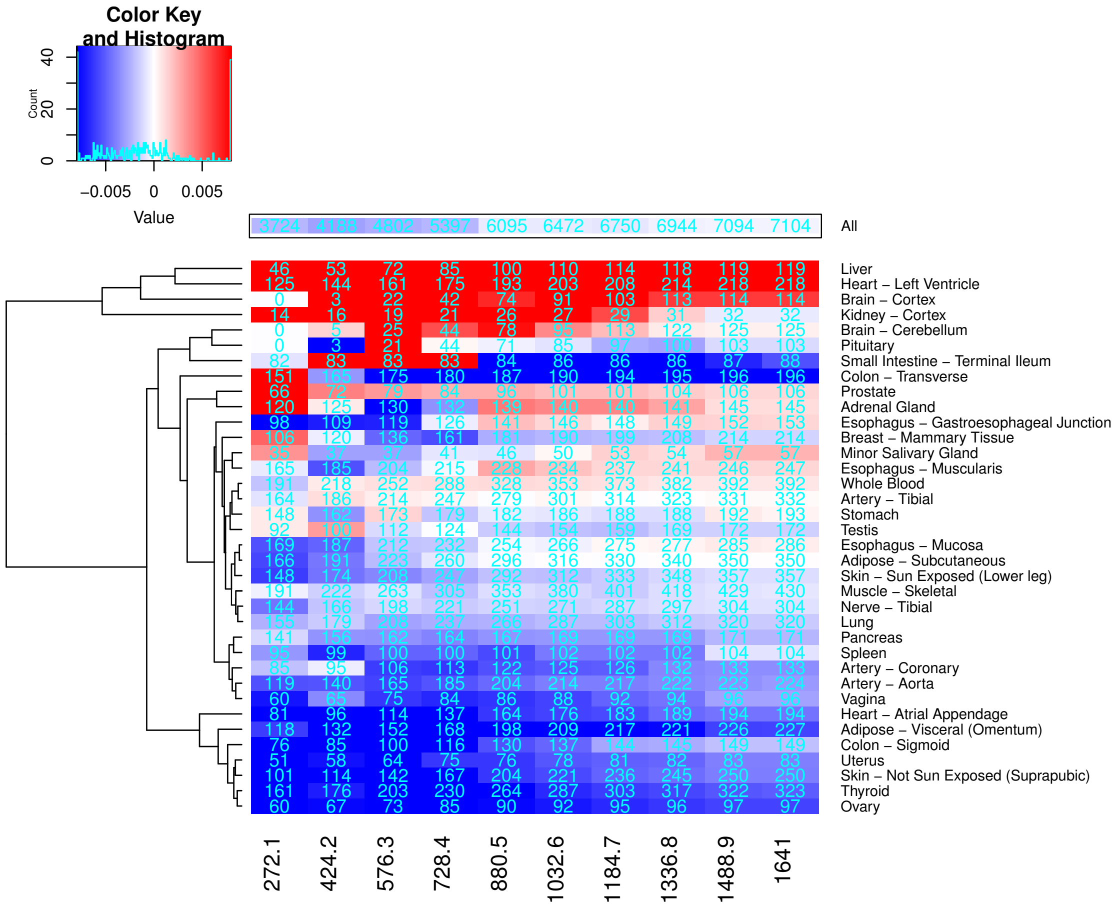

Supplementary Figure 17: Temporal changes of the concentration of mtRNA. Each cell of the heatmap represents the slope value (from decreasing represented in blue to increasing represented in red, see color key) of the linear regression considering samples up to a certain time *t* of PMI (x-categories) of each tissue (y-axis). The values in green represent the number of samples considered in each category. Tissues in the upper clusters (Liver, Kidney, Brain, etc.) show an increasing proportion of mitochondrial RNAs along the PMI, whereas the lower clusters (Lung, Skin, Nerve, Bladder, Pancreas, Spleen, Adipose, Artery, Ovary, Colon, Vagina, Thyroid, Uterus) exhibit declining mitochondrial transcriptional activities. See examples of two tissues (Liver and Ovary) with increasing and decreasing patterns in Figure 3.

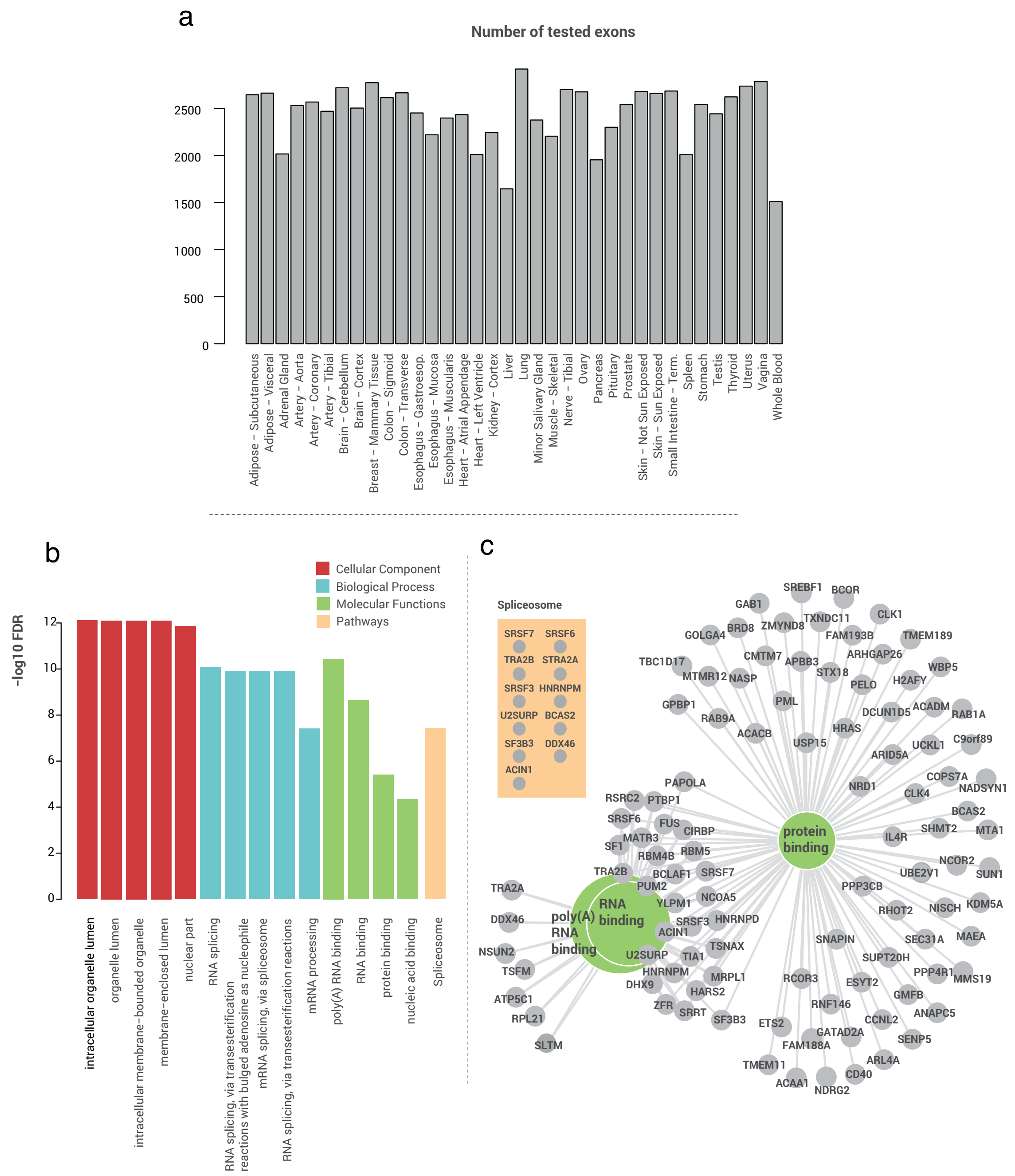

Supplementary Figure 18: Exon differential inclusion. a) Number of tested exons for association of PMI and exon inclusion levels (PSI) across the different tissues. Only exons with sufficient read coverage were considered for this analysis. (b) Functional analysis shows enrichment in mRNA splicing and processing functions. (c) Genes involved in the enriched functions.

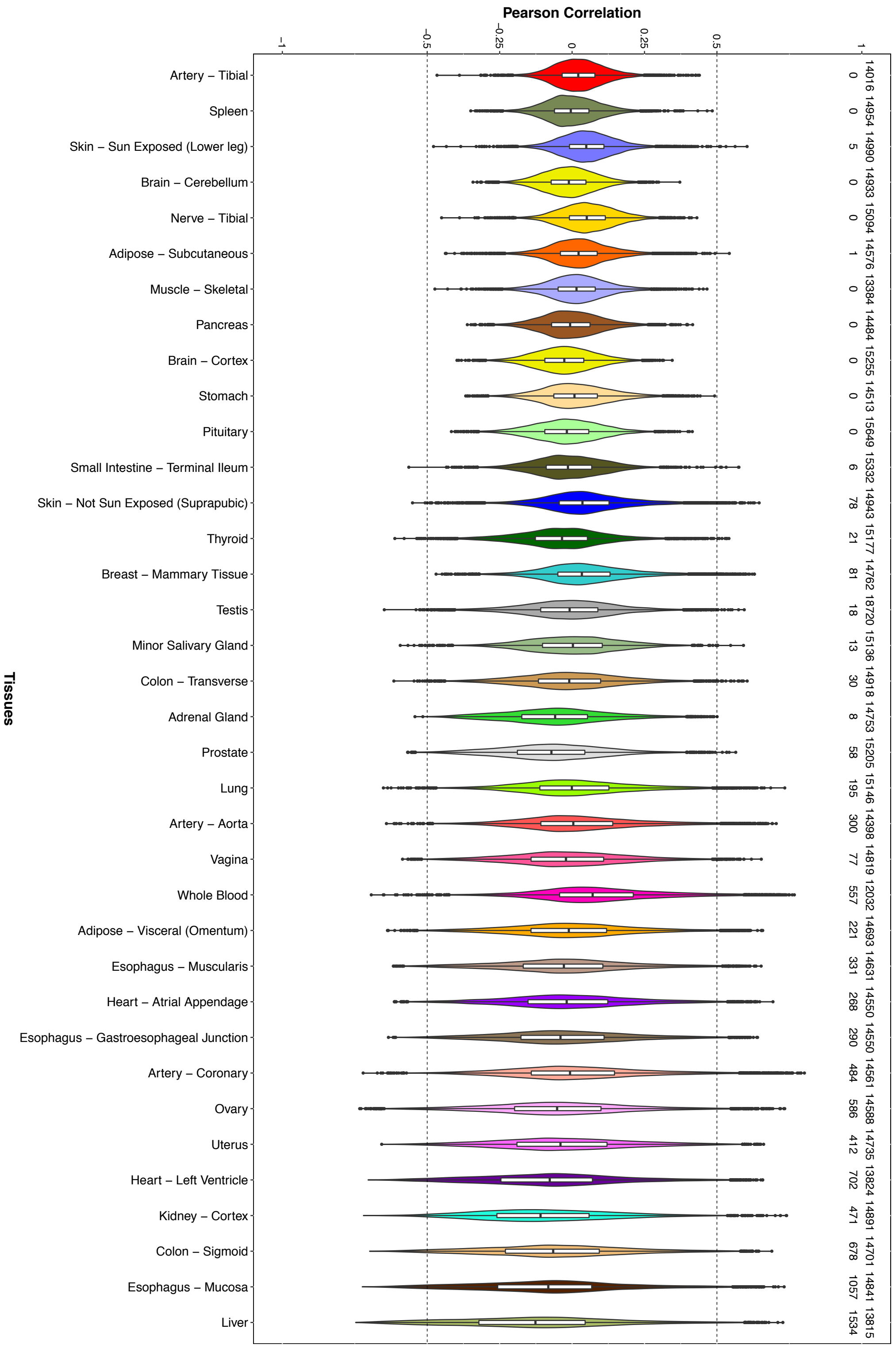

Supplementary Figure 19: Distribution of correlation values (Pearson) between Splicing Entropy and PMI. Splicing entropy is calculated for all genes with two or more transcripts based on the relative abundance of transcripts. Tissues were sorted by the interquartile range of the correlation values. For each tissue (right side) on the top row is presented the total number of tested genes and below the number of genes with a correlation deemed significant (|r| > 0.5 and FDR<5%). The number of significant genes is independent of tissue sample size.

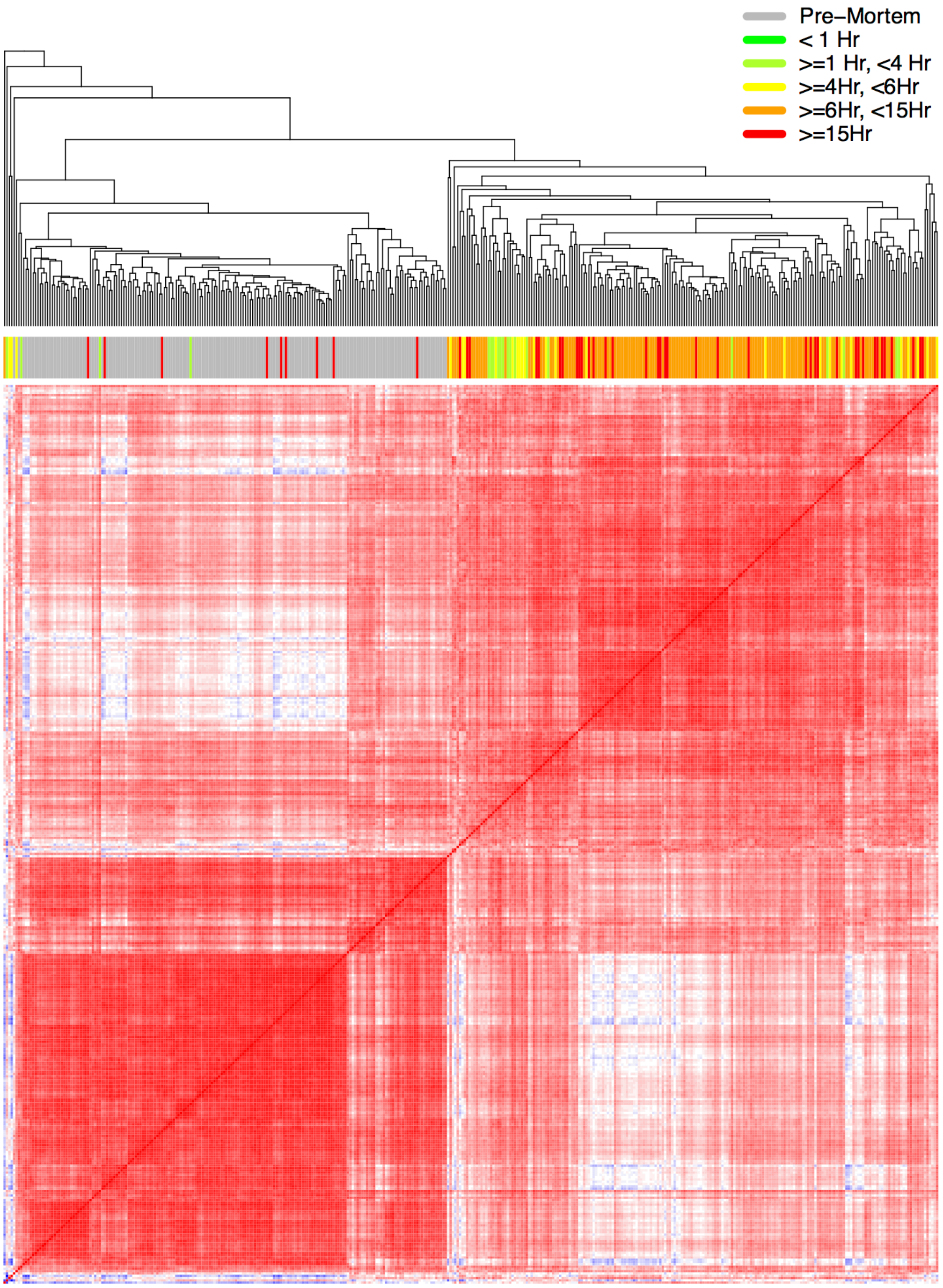

Supplementary Figure 20: Hierarchical Clustering and heatmap of pre- and postmortem Blood samples. The gene expression profiles of pre (gray) and post-mortem (interval colors) samples show a clear separation of these two groups of samples.

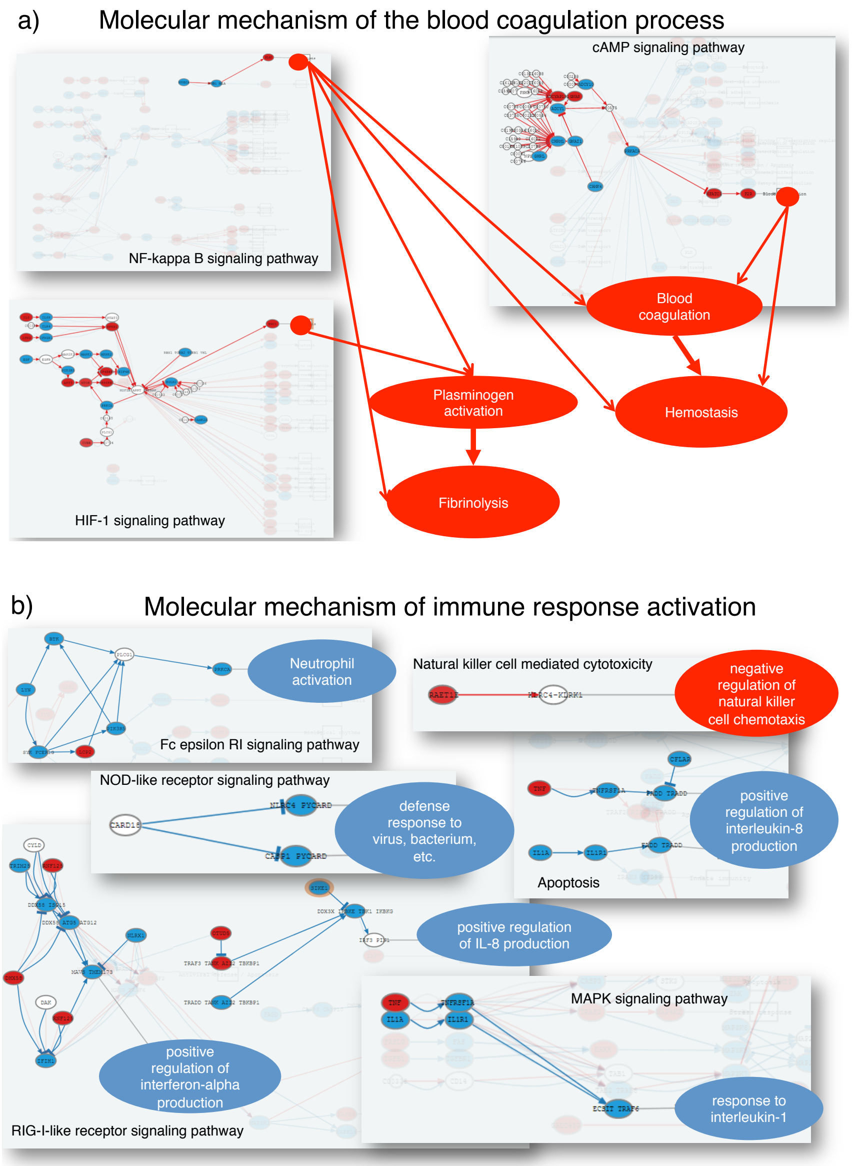

Supplementary Figure 21: Signaling pathways with changes in gene expression between Pre and Post-mortem samples. Functions in red indicate activated expression in post-mortem samples and deactivations in blue. a) Pathways involved in Blood coagulation b) Pathways involved in immune response.

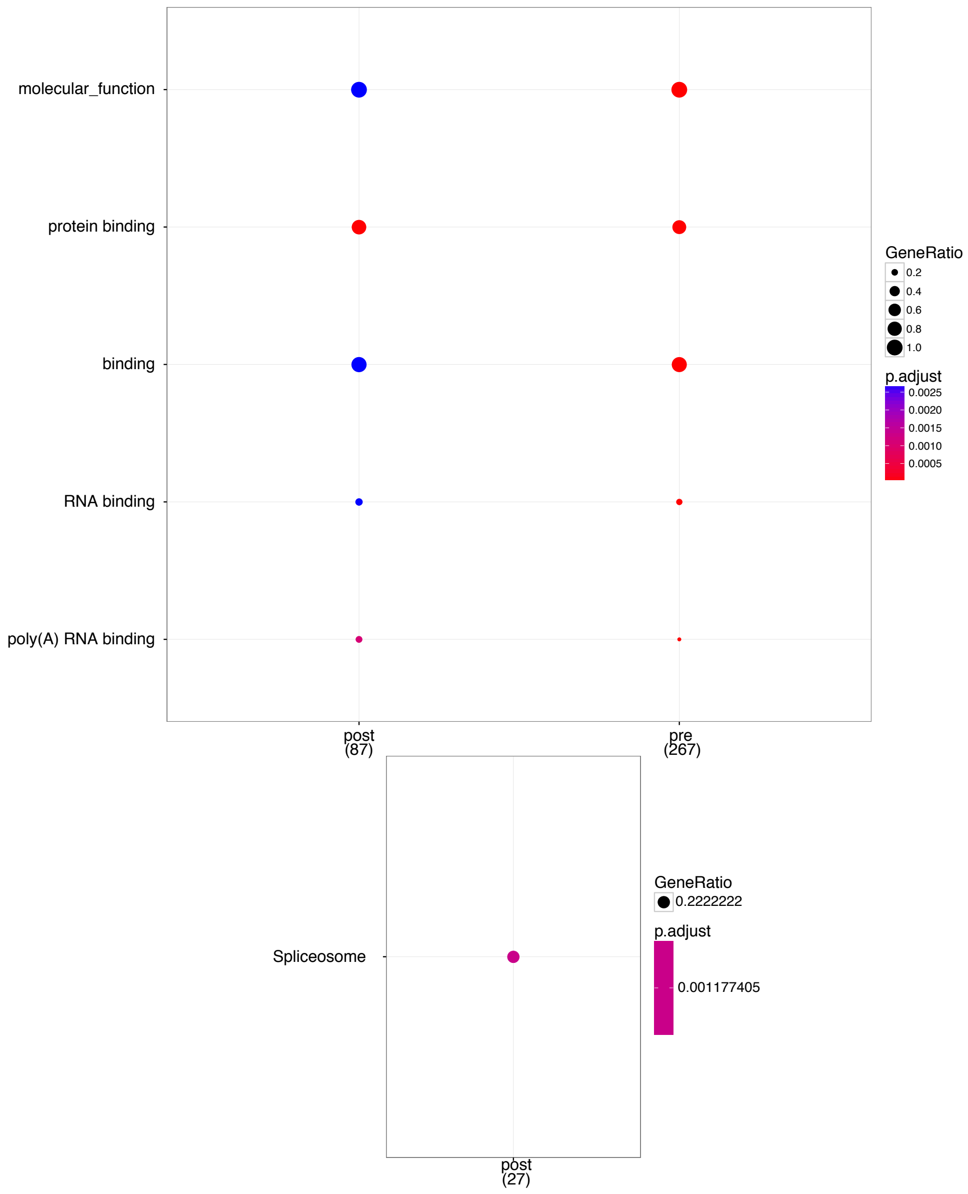

Supplementary Figure 22: GO and KEGG enrichment for exons differentially included in between Pre and Post-mortem Blood samples.

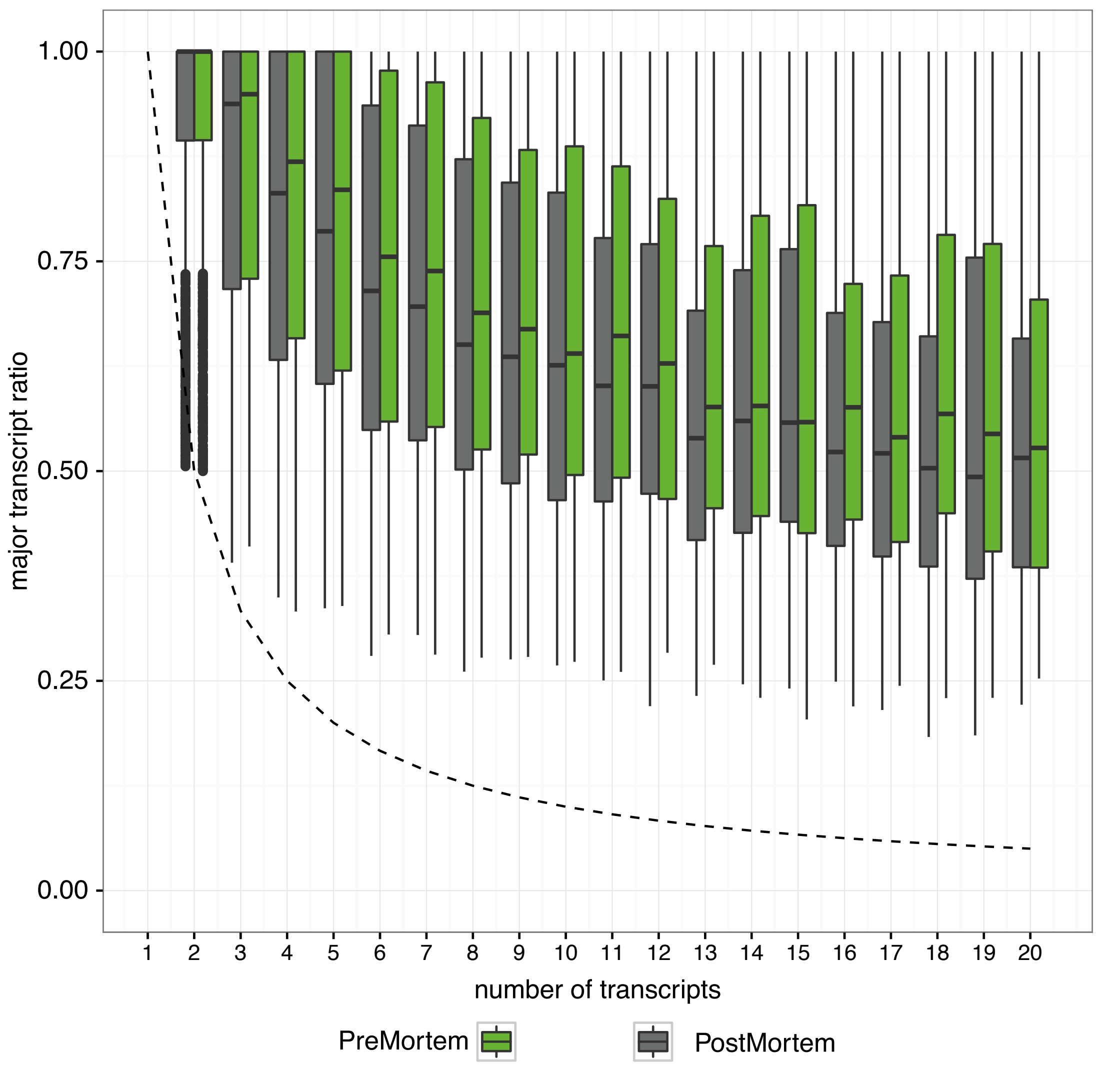

Supplementary Figure 23: Usage of the major transcript in Pre and Post-mortem Blood samples. The major transcript ratio is calculated as the ratio of expression of the most abundant transcript in the gene over the total expression of the gene.

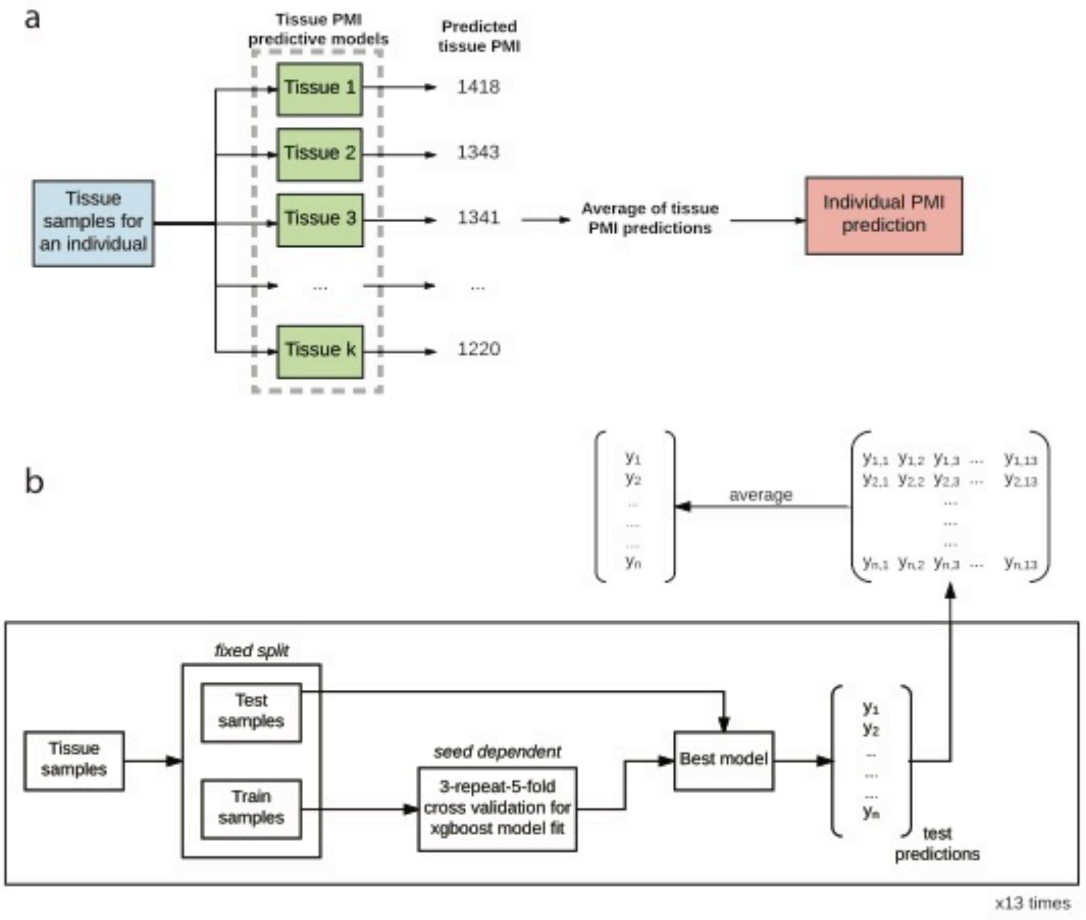

Supplementary Figure 24: Pipelines for PMI prediction. a) Pipeline for the individual PMI prediction procedure. For a given individual of the test set, we apply the fitted models over the available tissue samples in order to generate a PMI value for each tissue. For each of these predictions, we subtract the elapsed time since the start of the GTEx procedure in order to normalize, and then average the resulting values to obtain the individual PMI. b) Pipeline for tissue model generation using gradient boosted trees. First, we create a fixed split of individuals into training and test sets. For a given tissue, we perform 3-repeat-5-fold cross validation in order to select the best model, and we generate the predictions over the test set using this model. This process is repeated 13 times using different seeds to take into account the variation in the hyperparameter optimization process. The output is a matrix of n samples x 13 columns, where each column represents the tissue PMI prediction of all samples for each iteration. The final tissue PMI predictions will be taken as the row average of this matrix.

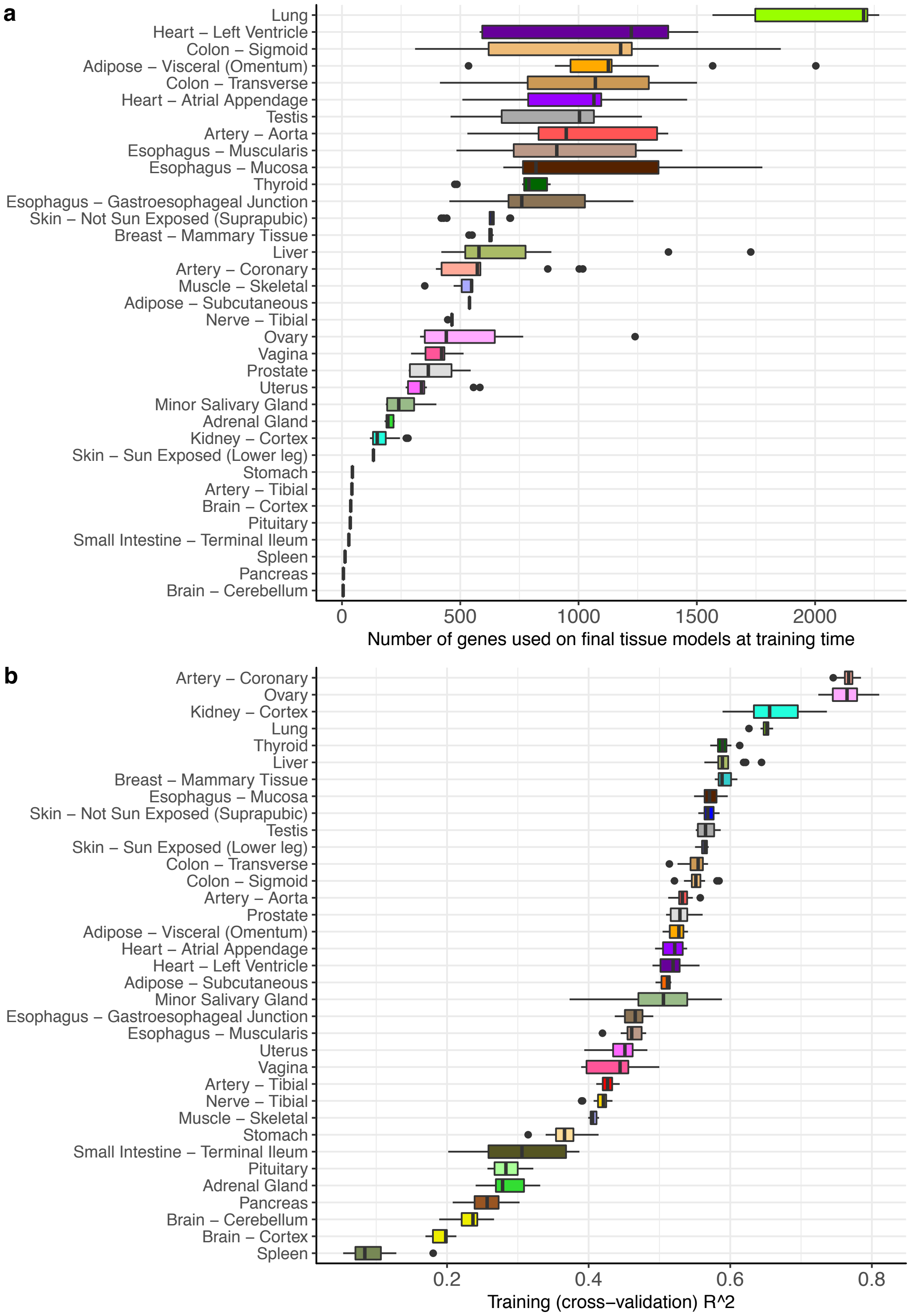

Supplementary Figure 25: Characteristics of the final models across different tissues. a) Variability of the number of genes used on each of the 13 final tissue models at training time, per tissue. b) Variability of R^2 values among the 13 final fitted models at training time, per tissue.

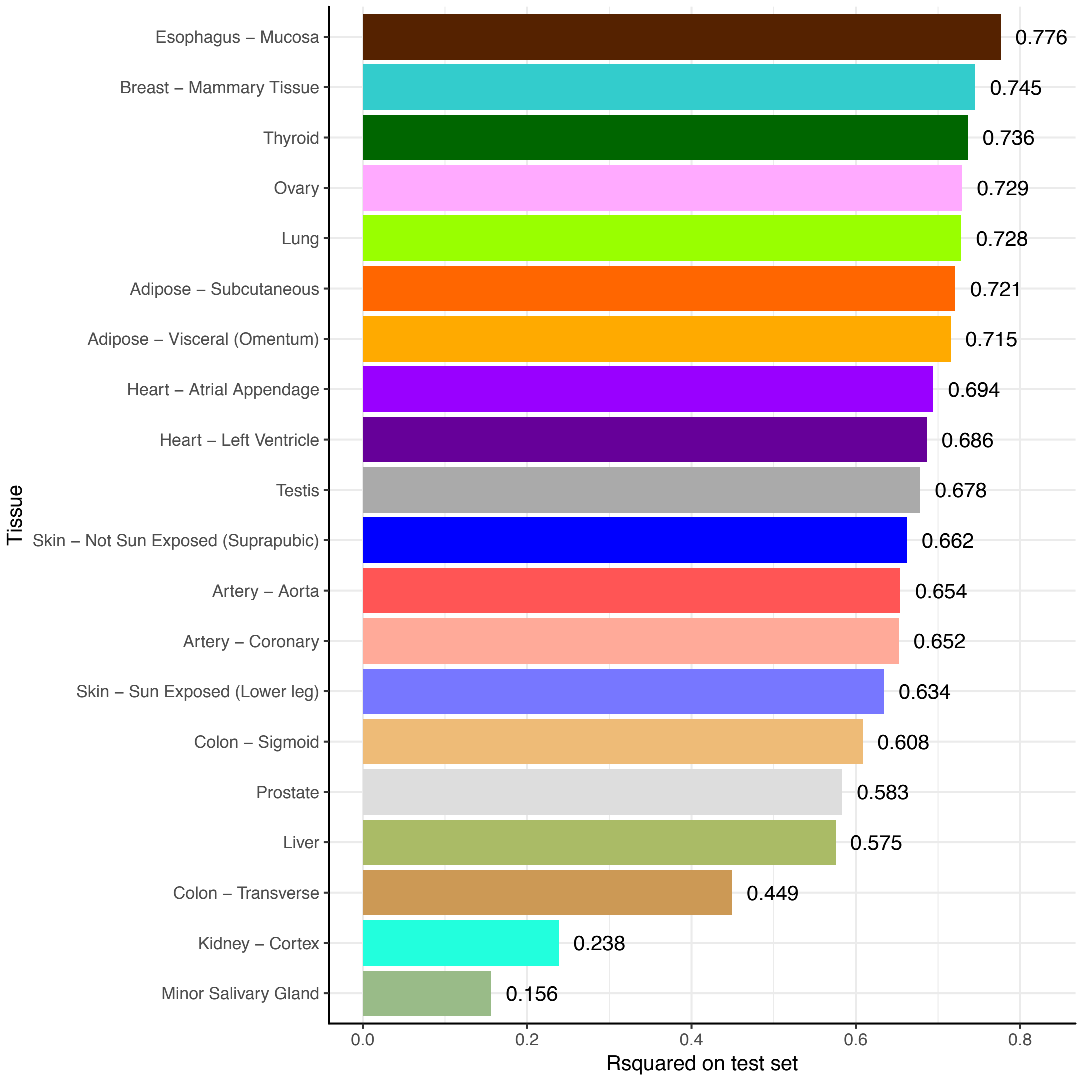

Supplementary Figure 26: R^2 of the regression between real and predicted tissue PMI on the test set. Only the 20 tissues selected in the training process (those with training $R^2 > 0.5$) are shown.

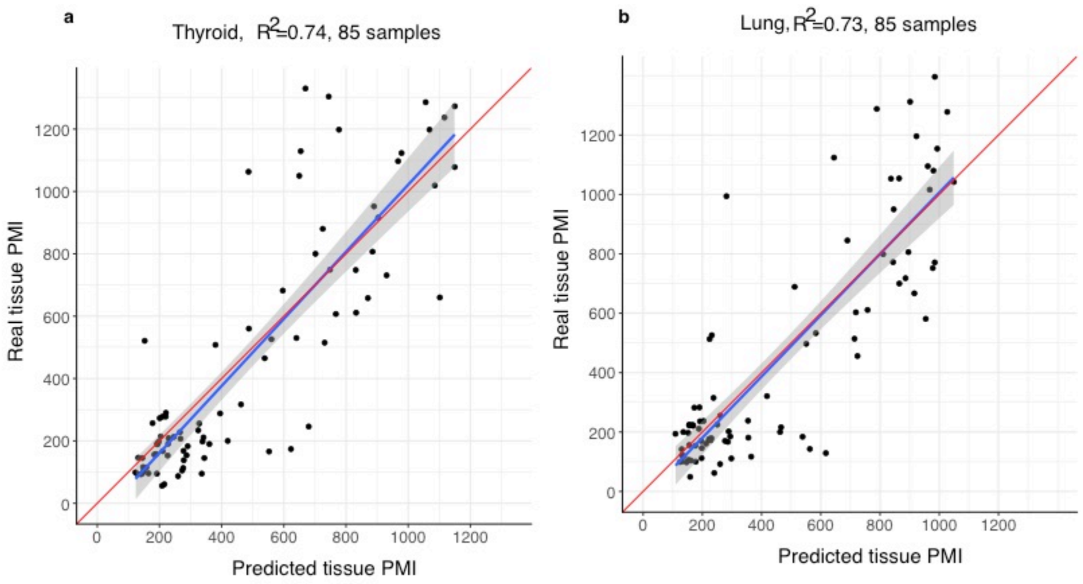

Supplementary Figure 27: Regression between real and predicted tissue PMI for the samples in the test set. R^2 values for a) Thyroid and b) Lung.

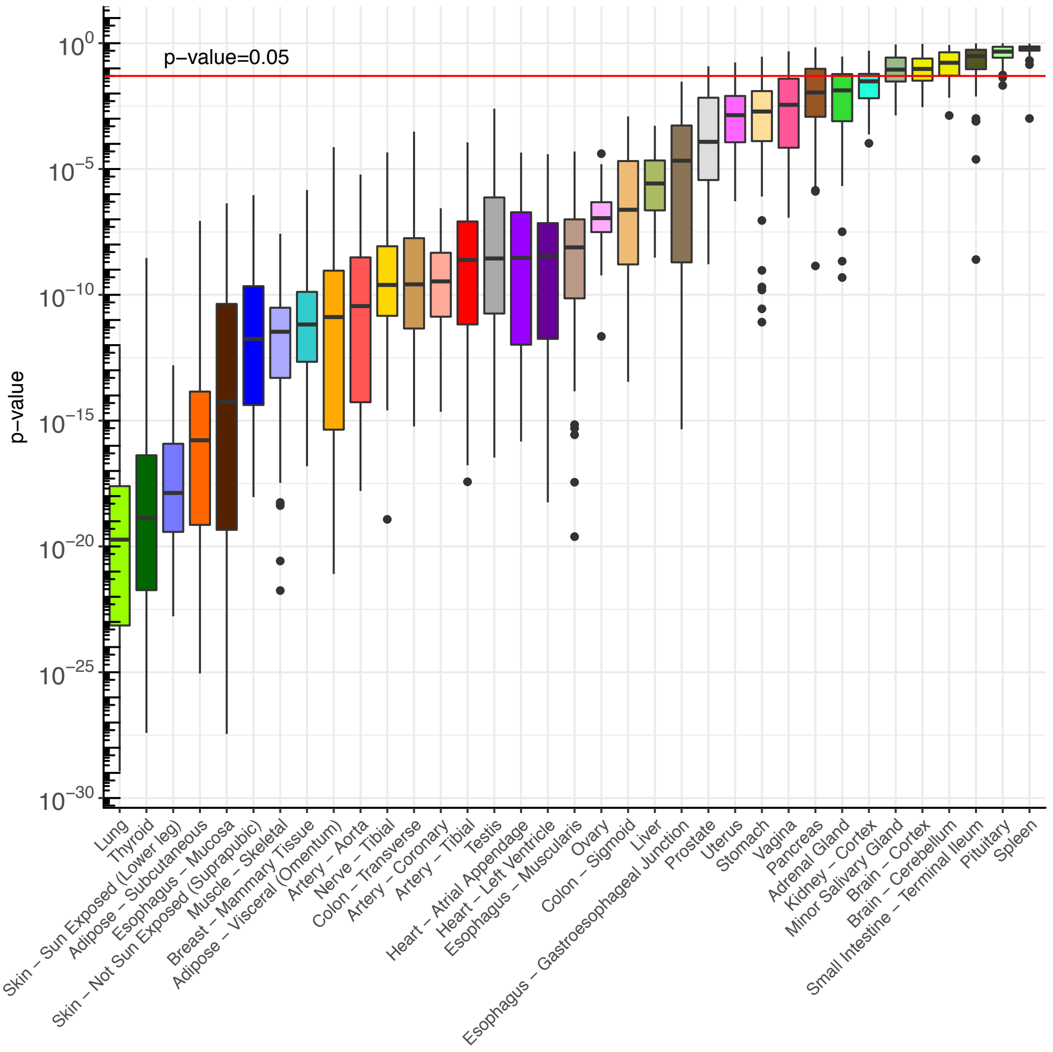

Supplementary Figure 28: Variability of the p-value of the F-test for the regression coefficient of real tissue PMI versus predicted tissue PMI in the 50-resamples experiment for tissue model stability.

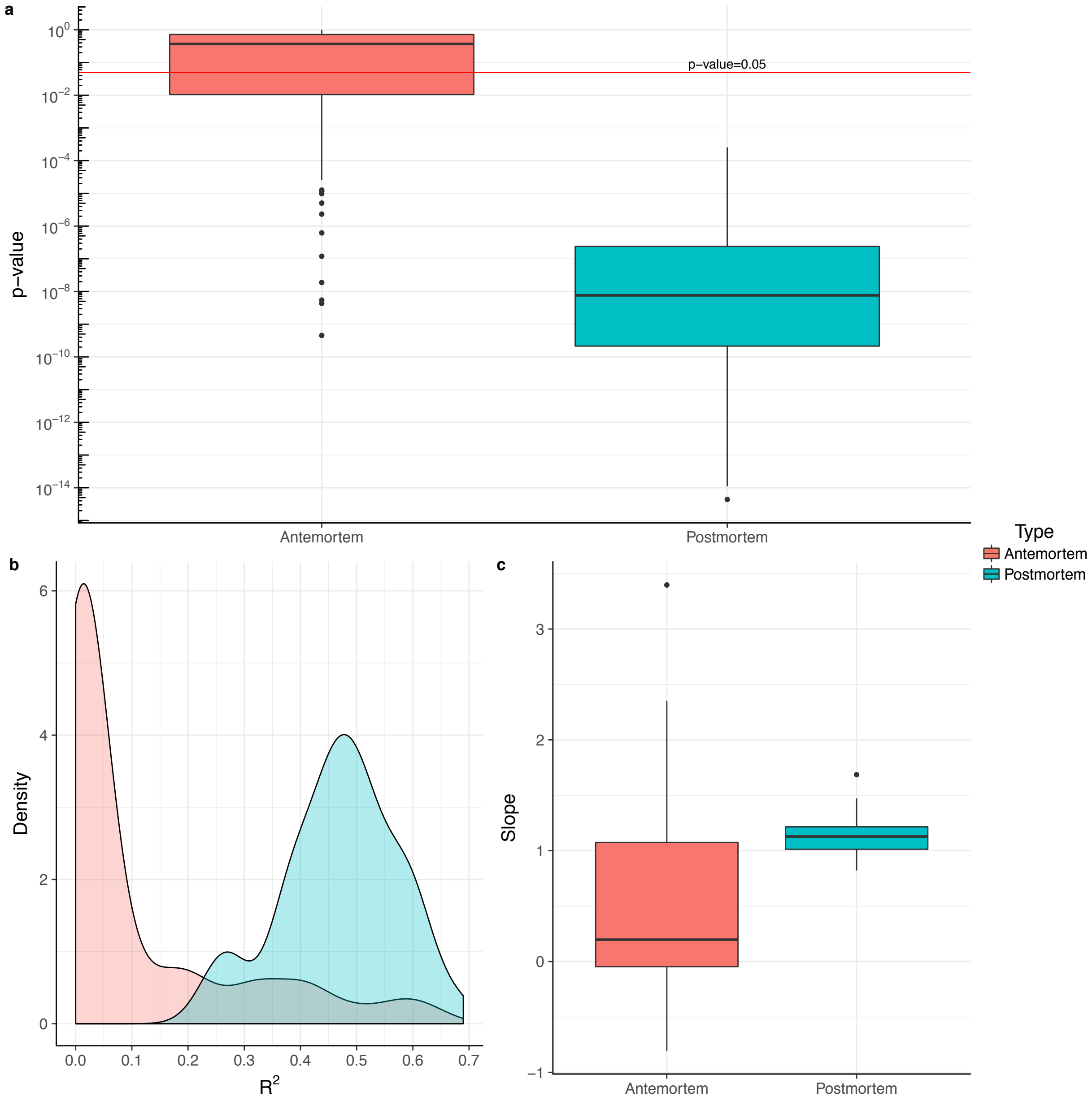

Supplementary Figure 29: Variability of the regression statistics of real vs. predicted Blood PMI, for a hundred repetitions of model fitting. Regression statistics calculated over pre-mortem and post-mortem samples. a) Distribution of p-values; b) Distribution of R2 values of the regression; c) distribution of regression slope values. The median p-value of the regression coefficient for post-mortem predictions was 10^{-8}, while it was near 1 for the pre-mortem predictions.

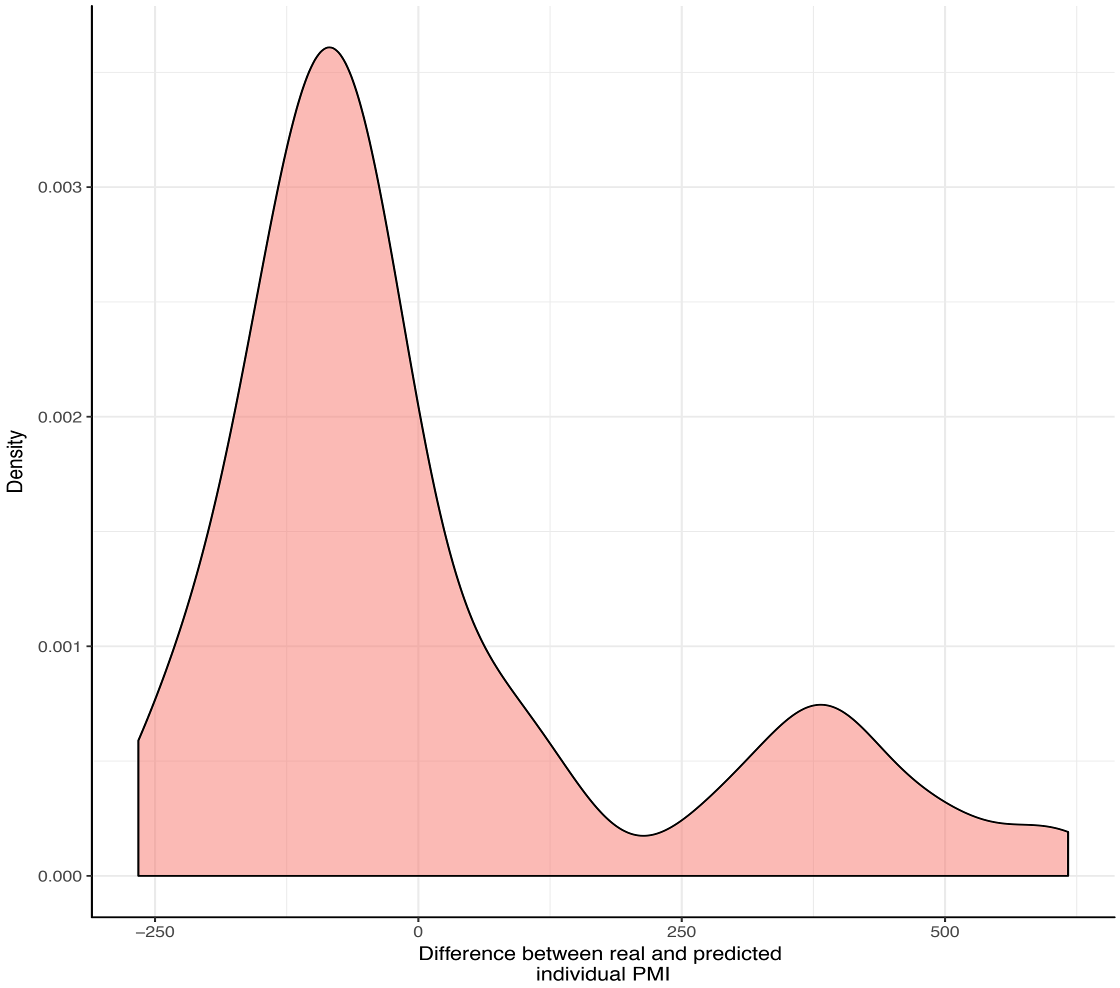

Supplementary Figure 30: Density of the individual PMI prediction error (signed difference between real and predicted individual PMI) on the test set. When predicting the final individual PMI, the median error is -63.75 minutes, while the mean error is 9.45 minutes.

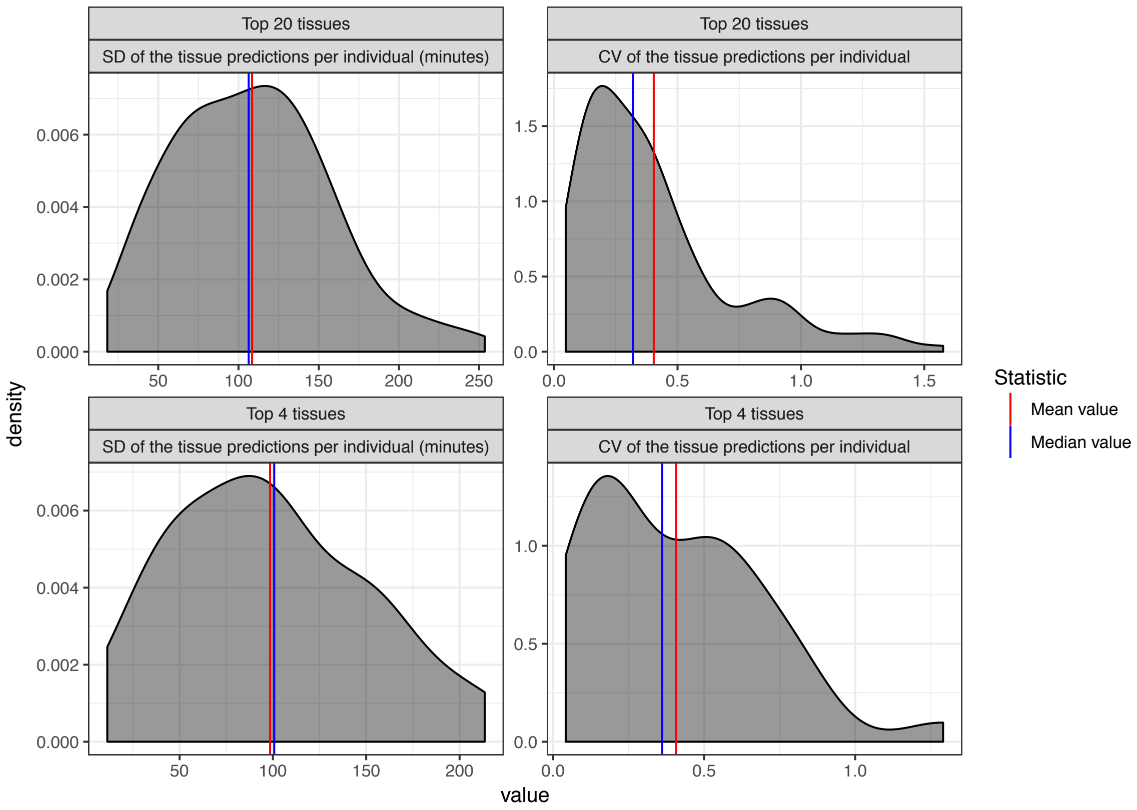

Supplementary Figure 31: Stability of the corrected tissue PMI predictions. Standard deviation (SD) and Coefficient of Variation (CV) for the models based on the top 20 tissues (SD min: 18.22, median: 106.3, mean: 108.5, max: 253.5; CV min: 0.05, median: 0.32, mean: 0.4, max: 1.58) and the best subset of 4 tissues (Adipose – Subcutaneous, Lung, Thyroid, and Skin (Sun Exposed) (SD min: 11.31, median: 100.7, mean: 98.51, max: 213.4; CV min: 0.04, median: 0.36, mean: 0.41, max: 1.29).

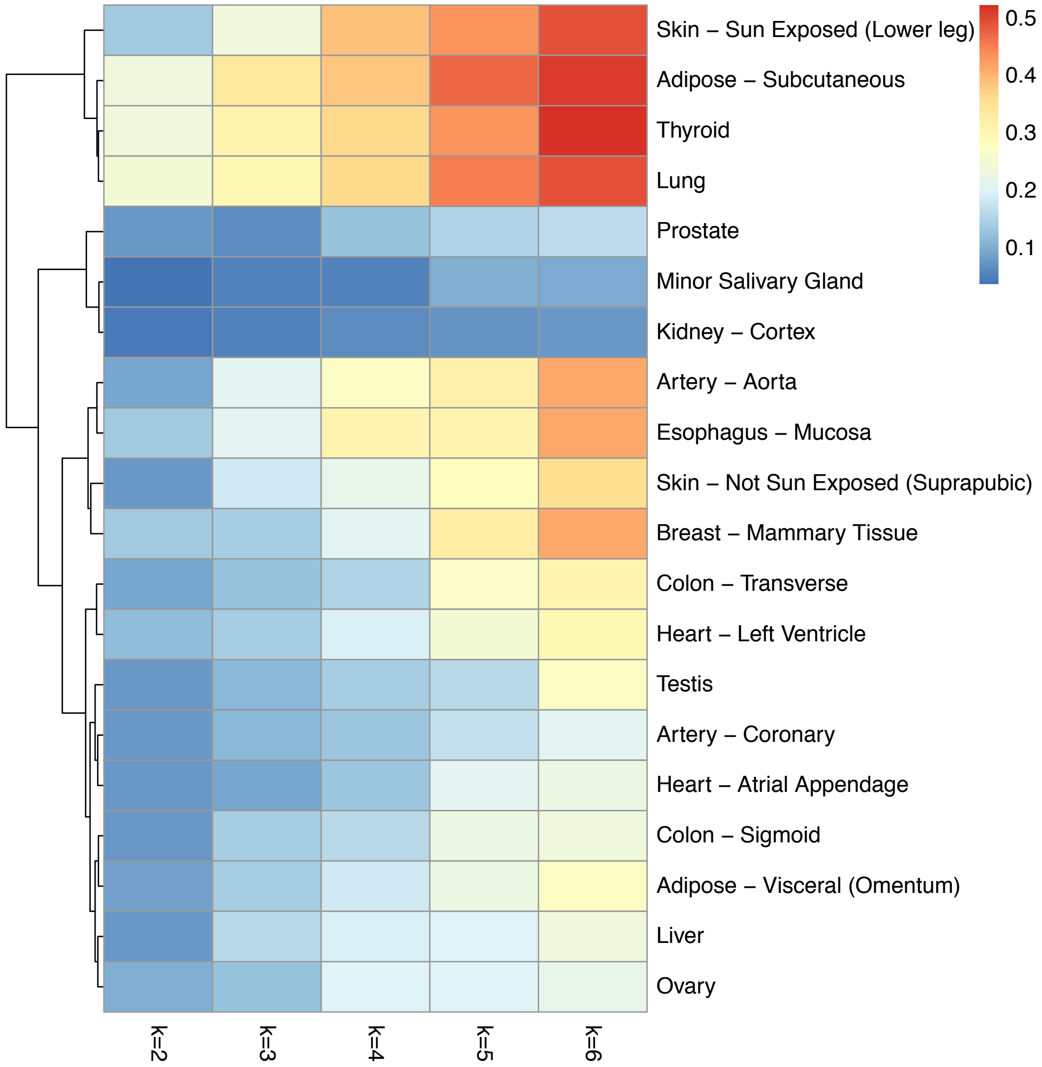

Supplementary Figure 32: Proportion of independent tissue presence in optimal sets of sizes k=2 to k=6. For each set size (i.e. each column), the proportion is calculated with respect to the number of individuals that had combinations of size k available.

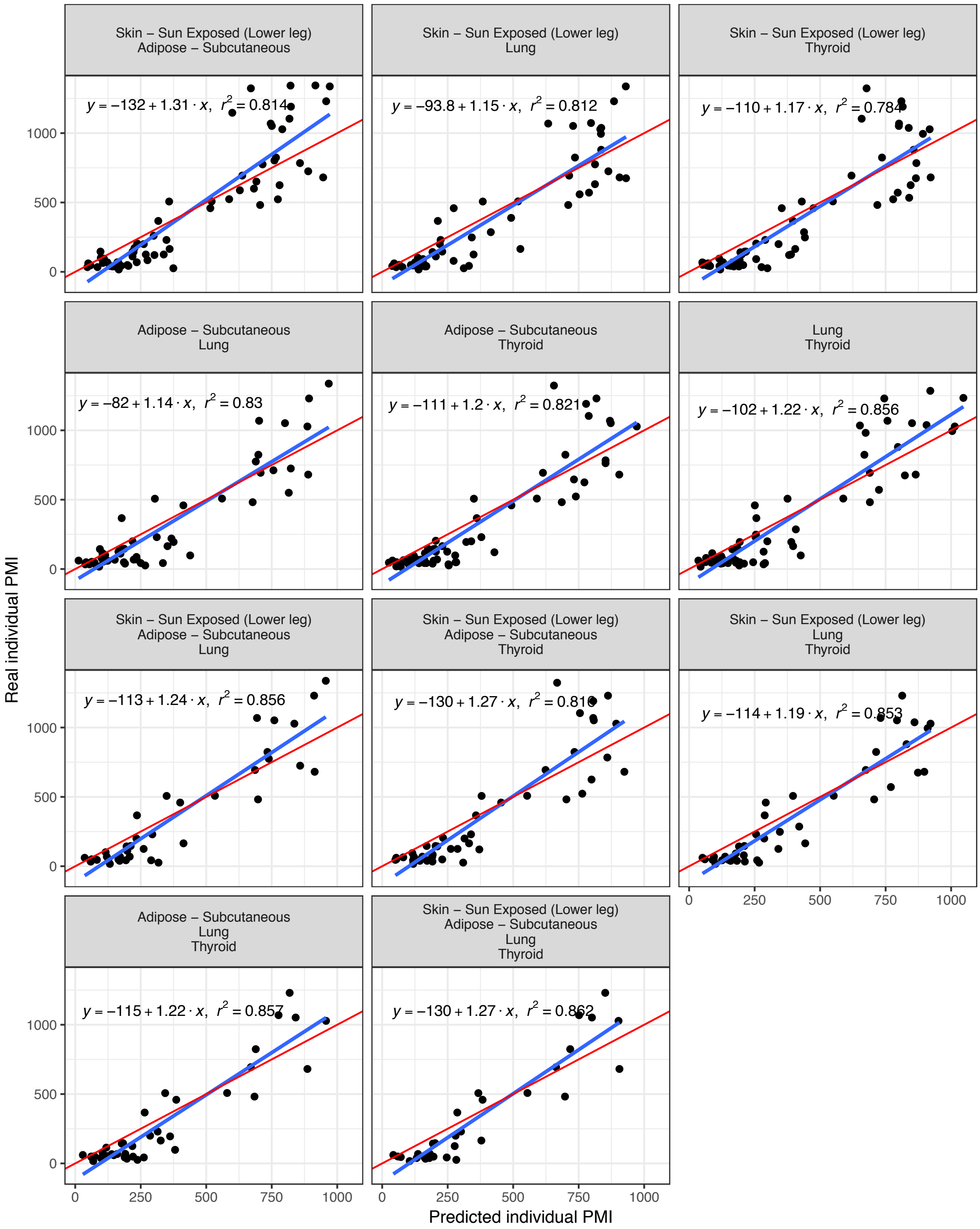

Supplementary Figure 33: Regressions of real vs. predicted individual PMI for all combinations. Size 2, 3 and 4 used in the four tissues (Adipose - Subcutaneous, Lung, Skin - Sun Exposed (Lower leg), Thyroid) selected from the optimal tissue combination analysis. Sample sizes from top left to bottom right: n=64, 55, 61, 53, 59, 60, 38, 46, 43, 41, 31.

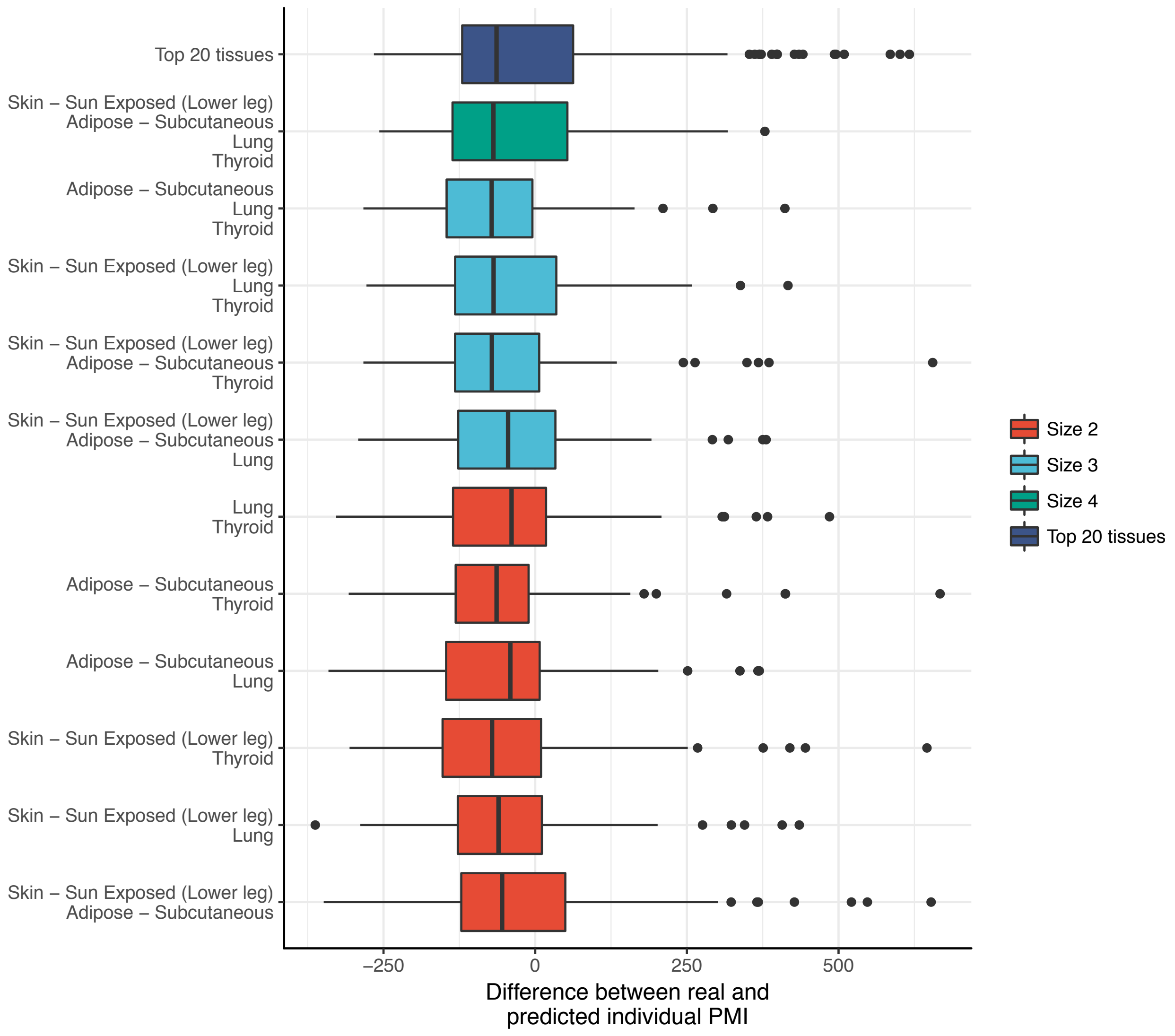

Supplementary Figure 34: Variability of the individual PMI prediction error (difference between real and predicted individual PMI) for all combinations of size 2, 3 and 4 using the four tissues (Adipose - Subcutaneous, Lung, Skin - Sun Exposed (Lower leg), Thyroid) selected from the optimal tissue combination analysis, and also compared with the top 20 tissues selected on the main prediction procedure.

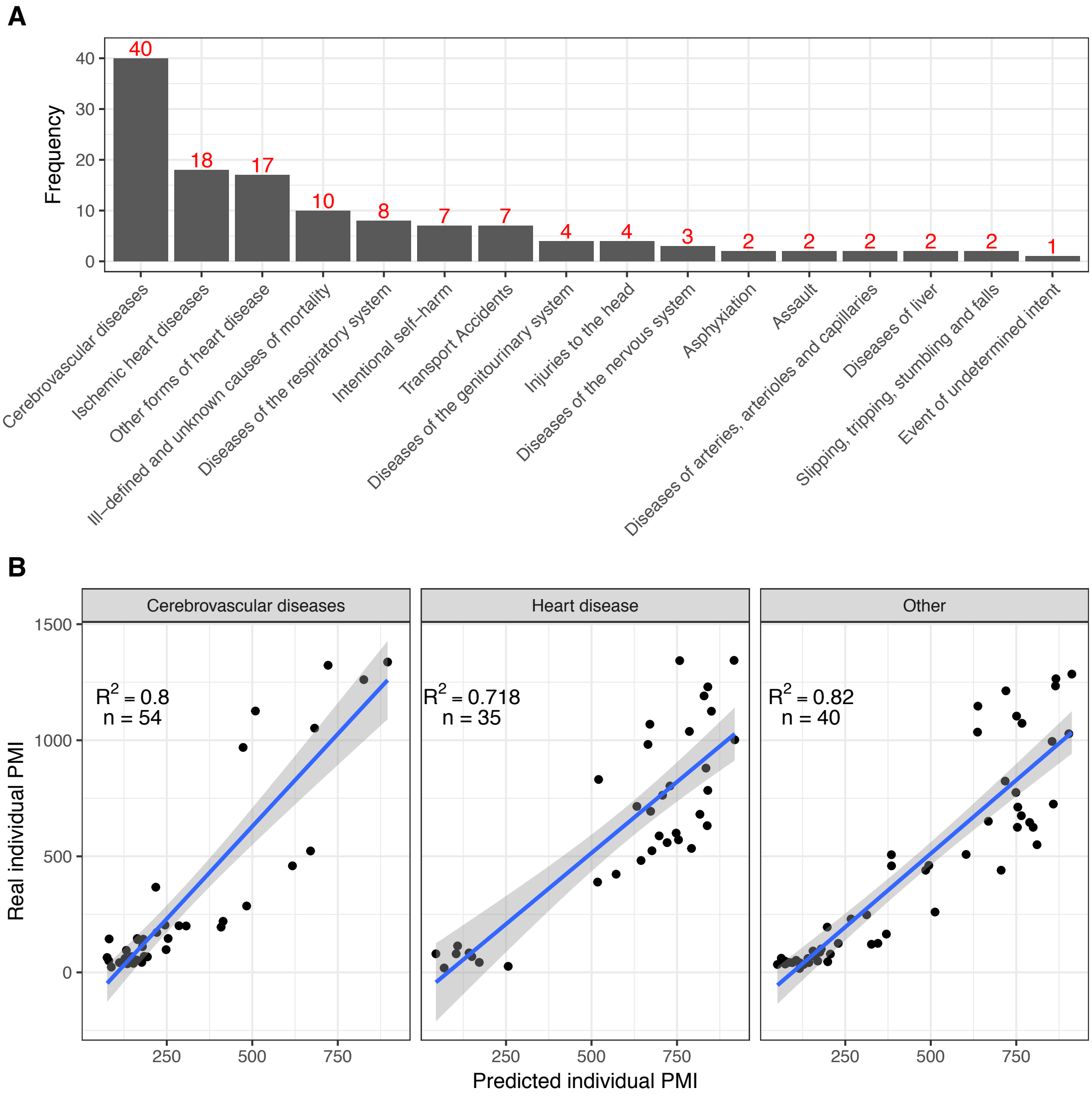

Supplementary Figure 35: Impact of cause of death on prediction performance. A) Distribution of the number of GTEx individuals in the test set according to the different causes of death. B) R^2 values for the real vs. predicted individual PMI prediction categorized by cause of death show no impact of the different death classes in the accuracy of the prediction.

A

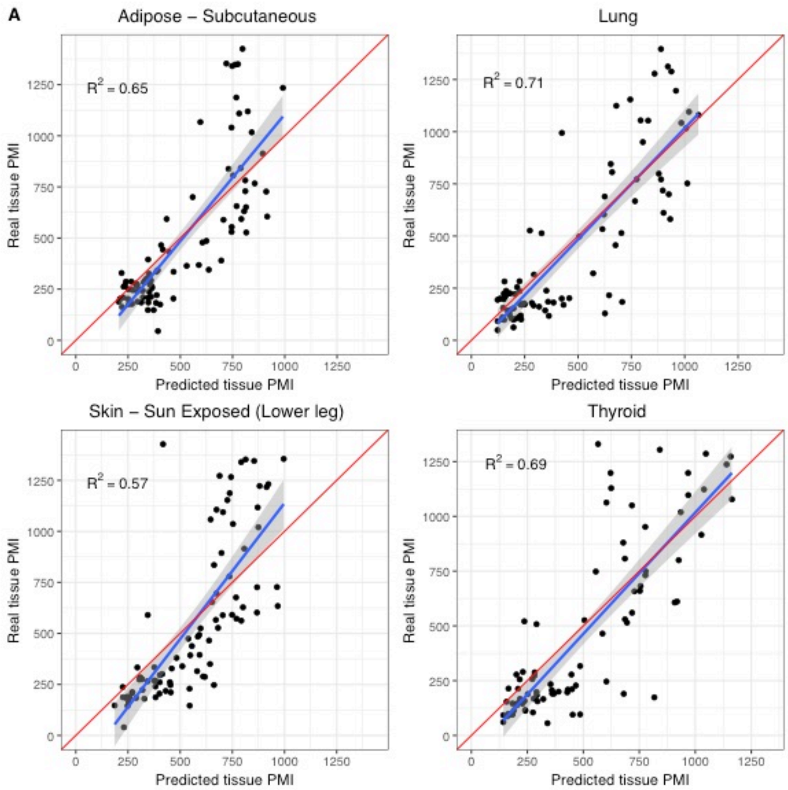

B

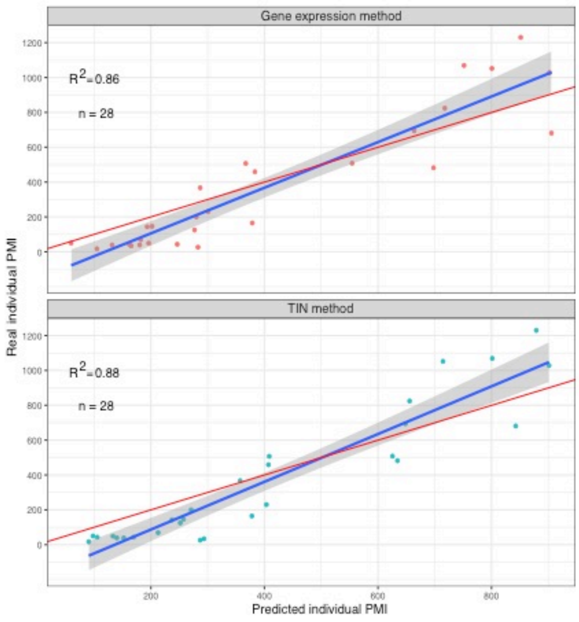

C

	TIN Method	Gene Exp. Method	Intersection
Lung	600	962	64
Adipose - Subcutaneous	944	538	63
Thyroid	964	698	75
Skin - Sun Exposed	1294	133	25

Supplementary Figure 36: Performance of the PMI prediction model based on TIN measure. A) R^2 values for the real vs. predicted tissue PMI. B) Comparison of the performance with the model based on gene expression for individual PMI prediction. C) Number of genes (with feature importance larger than 0.1, as computed by *xgboost*) included in the models based on TIN and gene expression measures and respective intersection.

Supplementary Note 1

Ischemic time (Post-Mortem Interval)

GTEx annotation provides three values for ischemic time described as following:

- **Total Ischemic time for a sample** (SMTSISCH, Minutes), Sample Ischemic Time: Interval between actual death, presumed death, or cross clamp application and final tissue stabilization.
- **Total Ischemic time for a donor** (TRDNISCH, Minutes), Donor Ischemic Time: Interval between actual death, presumed death, or cross clamp application and first tissue stabilization; A single donor-level ischemic time point at which the first tissue was collected for that donor.
- **Ischemic Time** (TRISCHD, Minutes), GTEX Procedure Start Time: Interval between actual death, presumed death, or cross clamp application and the start of the GTEx Procedure (Unit is Minutes).

The first value refers to the sample ischemic time while the last two refer to the ischemic time of the individual. For tissue analysis we used the SMTSISCH variable, while for the analysis in the section '*Prediction of the Post-mortem Interval from gene expression patterns across multiple tissues*' we used both TRISCHD and SMTSISCH. Throughout the text we will use the term Post-Mortem Interval (PMI) to refer to ischemic time and except if explicitly stated it refers to the sample ischemic time (SMTSISCH).

Hardy Scale Criteria

Death classification based on the 4-point Hardy Scale as in the GTEx annotation files:

1) Violent and fast death. Deaths due to accident, blunt force trauma or suicide, terminal phase estimated at < 10 min.
2) Fast death of natural causes. Sudden unexpected deaths of people who had been reasonably healthy, after a terminal phase estimated at < 1 hour (with sudden death from a myocardial infarction as a model cause of death for this category)
3) Intermediate death. Death after a terminal phase of 1 to 24 hours (not classifiable as 2 or 4); patients who were ill but death was unexpected
4) Slow death. Death after a long illness, with a terminal phase longer than 1 day (commonly cancer or chronic pulmonary disease); deaths that are not unexpected
0) Ventilator Case. All cases on a ventilator immediately before death.

Supplementary Note 2

PMI regression model

To calculate correlation of gene expression with PMI the following procedure is applied to each of the selected tissues. For this analysis we only considered genes with a mean RPKM expression value (before further normalization) across samples from a given tissue greater than 0.5.

```
Function: CorrelationExpressionPMI_with_Covariates
Input: tissue.name
        # retrieve the sample ids from the input tissue
        samples = getSamplesFromTissue(tissue.name);
        # variables.matrix is the table with sample annotation
        # get the PMI for the samples
        pmi.vals = getPMI(variables.matrix, samples);
        # get the RPKM values for genes in the selected samples
        # log transform and normalize
        rpkm.norm.matrix = getExpressionNormalized(rpkm.matrix, samples);
        For each gene in rpkm.norm.matrix, do:
                gene_expression = getExpression(rpkm.norm.matrix, gene)
                # select genes with minimal (raw RPKM) expression
                If mean_raw_gene_expression(gene) > 0.5, do:
                        # fit a linear model
                        # matrix.selectedCovariates selected covariates table
                        reg = lm(gene_expression ~ matrix.selectedCovariates)
                        # extract the residuals of the model as the expression phenotype
                        gene_expression.resid =  residuals(reg)
                        # calculate correlation with PMI and gene expression residuals
                        R = correlation(gene_expression.resid, pmi.vals)
                        # output results
                        write (tissue_name, gene, R.pearson, R.pearson_pval)
```

Supplementary Figure 37: Pseudo-code for the calculation of the correlation between PMI and gene expression considering covariates.

Function getSamplesFromTissues returns the list of all the samples corresponding to the tissue in analysis. Function getPMI retuns the PMI values for the list of samples passed to the function. Function getExpression retrieves a matrix of RPKM values for all genes and for the samples passed to the function. The rpkm values are first log2 transformed ($\log_2$(rpkm + 0.5)); these values are then normalized with the function normalize.quantiles from packages *preprocessCore*. mean_raw_gene_expresion correspond to the transformation of the gene expression values to the natural scale and removing the pseudocount ($2^{gene\_expression}$ - 0.5).
To compute the correlation values of gene expression and PMI without the covariates a procedure similar to the above was used where Pearson correlation is obtained between gene expression and PMI values.

```
Function: CorrelationExpressionPMI
Input: tissue.name
        # retrieve the sample ids from the input tissue
        samples = getSamplesFromTissue(tissue.name);
        # variables.matrix is the table with sample annotation
        # get the PMI for the samples
        pmi.vals = getPMI(variables.matrix, samples);
        # get the RPKM values for genes in the selected samples
        # log transform and normalize
        rpkm.norm.matrix = getExpressionNormalized(rpkm.matrix, samples);
        For each gene in rpkm.norm.matrix, do:
                gene_expression = getExpression(rpkm.norm.matrix, gene)
                # select genes with minimal (raw RPKM) expression
                If mean_raw_gene_expression(gene) > 0.5, do:
                        # calculate correlation with PMI and gene expression
                        R = correlation(gene_expression, pmi.vals)
                        # output results
                        write (tissue_name, gene, R.pearson, R.pearson_pval)
```

Supplementary Figure 38: Pseudo-code for the calculation of the correlation between PMI and gene expression without including covariates.

To calculate the number of genes with significant p-value we performed multiple testing adjustments with Benjamini-Hochberg method[1] in both analyses above.

Non-linear temporal differential expression

```
Function: detectTimeIntervalChanges
Input: tissue.name
        # retrieve the gene expression for the given tissue with normalized and transformed RPKM values
        tissue.expr = prepareTissueExpressionTable(tissue.name)
        # get the PMI for the samples
        tissue.pmi = getTissuePMI(tissue.name)

        for each gene in tissue.expr:
                gexp = tissue.expr[gene,]
                # do the Z-score normalization for the gene
                gexp_zscore = ZSore(gexp)
                (T1, T2, T3, T4, T5) = getExpressionByInterval(gexp_zscore, tissue.pmi)
                # calculate median values
                (T1_m, T2_m, T3_m, T4_m, T5_m) = median(T1, T2, T3, T4, T5)
                # test every two consecutive time intervals
                if(len(T_i)> 5 and len(T_i+1)> 5){
                        pval(i, i+1) = wilcox.test (T_i, T_i+1)
                        fold_change(i, i+1) = log2(median(T_i) / median(T_i+1))
                        testTable.store(gene, pval(i,,i+1), fold_change(i, i+1))
                }

        # return computed values
        return testTable

# call the above function and filter for pval < 0.05 and fold_change(fc) > 2
table = detectTimeIntervalChanges(tissue)
filter(table, pval < 0.05, fc>2)
```

Supplementary Figure 39: Pseudo-code for the calculation differential gene expression across PMI intervals.

Supplementary Note 3

Signaling pathway models

hiPathia[2], is a tool for the interpretation of the consequences of the combined changes of gene expression levels and/or genomic mutations in the context of signaling pathways. hiPathia transforms uninformative gene expression and/or genomic variation data into signaling circuit activities, which carry information on the different cell functionalities triggered by them. Such signaling activities not only account for the underlying molecular mechanisms of diseases or the mode of action of drugs but they can also be used as mechanistic features for the prediction of complex phenotypes.

This methodology attempts to directly transform gene expression values into measurements of cell functional activities. To achieve so, it uses signaling pathways, which represent how the cell triggers different actions in response to diverse stimuli. We call each sub-pathway that connects one (or more) receptor protein to a unique effector protein as circuit. Of notice, each effector protein triggers one or several well-defined cell functions (e.g. proliferation, lipid biosynthesis, etc.). Briefly, normalized gene expression levels are used as proxies of protein activity and then the intensity of signal that would arrive to the end of the circuit is calculated. In this way, a vector of activities (functional activities) for each individual is calculated. Then, conventional statistical tests are applied to compare pre and post mortem activities and look for significant differentially activated functions. Any functional activity is the result of the collective activation/deactivation of genes within each individual. Therefore, this method captures the interactions among genes in each individual. In addition, functions are associated to phenotypes, not gene to phenotypes and then to functions. If a phenotype is caused by a particular function, the direct association will be stronger than if this is obtained through the pieces of the function (the genes), see Supplementary Fig. 40. Supplementary Fig. 21 and Figure 6d shows examples of signaling pathways with altered activity between pre and post-mortem Blood samples.

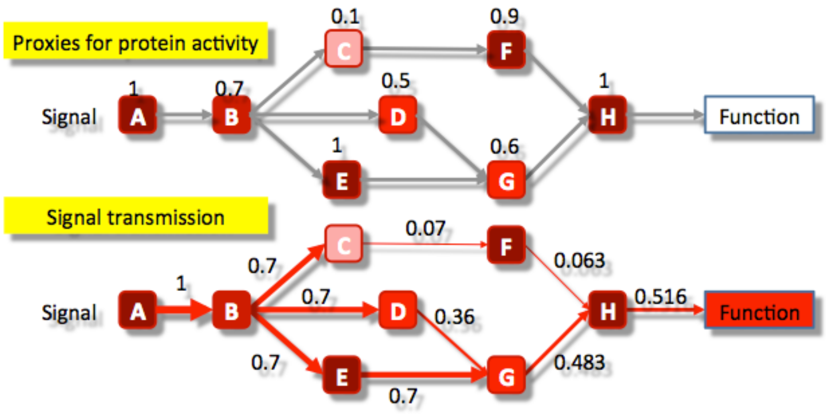

Supplementary Figure 40: Representation of signaling pathways by hiPathia.

One of the peculiarities of the methodology is that the activity values can be calculated at different levels. It can be calculated from i) one receptor to one effector,

ii) all the receptors that activate one effector and iii) all the receptors that activate one function. See Supplementary Fig. 41.

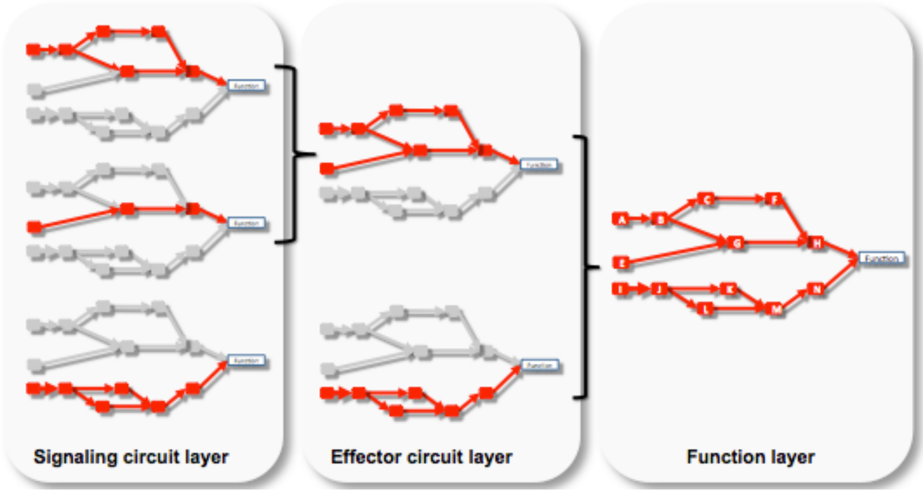

Supplementary Figure 41: Representation of different types of effectors in the signaling pathways.

Significant circuits associated to post-mortem times were obtained by fitting a linear model and were summarized by the median value across samples per circuit and time points. Then, the five main clusters of circuits obtained from a hierarchical clustering analysis were plotted as a functional activity progression across time points (Figure 6c).

Tables

Information	Table file
RNA-seq quality metrics from RNA-SeQC	GTEx_Analysis_2015-01-12_RNA-seq_RNA-SeQCv1.1.8_metrics.tsv
Samples attributes and annotation	GTEx_Analysis_2015-01-12_Annotations_SampleAttributesDS.txt
Subject phenotype	GTEx_Analysis_2015-01-12_Annotations_SubjectPhenotypesDS.txt
Correspondence between subject identifier and associated samples identifiers	GTEx_Analysis_2015-01-12_Annotations_SubjectSampleMappingDS.txt
Gene RPKM values	GTEx_Analysis_2015-01-12_RNA-seq_RNA-SeQCv1.1.8_gene_rpkm.gct.gz
Gene read count values	GTEx_Analysis_2015-01-12_RNA-seq_RNA-SeQCv1.1.8_gene_reads.gct.gz

Supplementary Table 1: Table files obtained dbGap corresponding to the data used in this analysis.

Tissue	Mean.MappedReads	Sd.MappedReads	Mean.MapRate	Sd.MapRate
Adipose - Subcutaneous	74148022.54	19673421.1	0.9	0.11
Adipose - Visceral (Omentum)	71745410.7	13256391.76	0.93	0.04
Adrenal Gland	75024613.5	16968579.31	0.94	0.02
Artery - Aorta	72952257.38	15176043.53	0.94	0.02
Artery - Coronary	75910465.55	30976060.6	0.94	0.02
Artery - Tibial	72328558.5	20423255.6	0.88	0.12
Bladder	62098077.55	9956866.7	0.95	0.01
Brain - Amygdala	77240528.33	23754643.75	0.87	0.13
Brain - Anterior cingulate corte	84259026.79	26728581.37	0.92	0.07
Brain - Caudate (basal ganglia)	80499989.36	23706630.13	0.88	0.13
Brain - Cerebellar Hemisphere	83837092.01	23420022.12	0.91	0.07
Brain - Cerebellum	75461669.57	17586885.15	0.9	0.07
Brain - Cortex	78201090.37	18041290.6	0.91	0.06
Brain - Frontal Cortex (BA9)	79521778.25	24970539.67	0.91	0.06
Brain - Hippocampus	75836788.23	20608371.04	0.89	0.12
Brain - Hypothalamus	81432278.46	40268512.85	0.9	0.11
Brain - Nucleus accumbens (ba	81741876.53	22635487.34	0.91	0.08
Brain - Putamen (basal ganglia)	78369791.9	25342085.56	0.9	0.11
Brain - Spinal cord (cervical c-1	76741078.39	21803607.6	0.88	0.15
Brain - Substantia nigra	75078654.97	20194765.43	0.88	0.13
Breast - Mammary Tissue	73651650.77	16236337.04	0.92	0.05
Cells - EBV-transformed lymph	87003019.58	20922707.41	0.93	0.02
Cells - Transformed fibroblasts	78008897.56	18265595.92	0.95	0.01
Cervix - Ectocervix	66670153	9487919.88	0.94	0.01
Cervix - Endocervix	61984002.8	5860888.79	0.94	0.01
Colon - Sigmoid	72908119.27	15542673.18	0.94	0.02
Colon - Transverse	72669549.19	15446082.3	0.93	0.01
Esophagus - Gastroesophageal	70880315.88	14638558.65	0.94	0.02
Esophagus - Mucosa	73463793.01	17405789.01	0.94	0.02
Esophagus - Muscularis	74126767.28	22832416.42	0.94	0.02
Fallopian Tube	67282165.67	15677759.06	0.94	0.01
Heart - Atrial Appendage	76413395.71	17588670.31	0.93	0.05
Heart - Left Ventricle	81251441.83	22794459.2	0.88	0.12
Kidney - Cortex	77831889	16382983.82	0.93	0.02
Liver	73836809.28	19941562.85	0.93	0.02
Lung	75358661.46	21401182.66	0.88	0.12
Minor Salivary Gland	73818490.3	14078929.7	0.93	0.02
Muscle - Skeletal	76082220.97	20388774.83	0.89	0.12
Nerve - Tibial	73244172.74	19828995.45	0.89	0.12
Ovary	72646461.51	15805258.76	0.93	0.02
Pancreas	71176490.65	15092710.89	0.89	0.04
Pituitary	72380284.96	16495091.13	0.92	0.05
Prostate	70603767.35	16658506.48	0.92	0.04
Skin - Not Sun Exposed (Suprap	71386698.78	14488852.56	0.93	0.04
Skin - Sun Exposed (Lower leg)	71141915.79	17626644.73	0.89	0.12
Small Intestine - Terminal Ileur	74029153.44	13364538.45	0.93	0.02
Spleen	68618520.02	13900946.44	0.92	0.02
Stomach	71507346.56	14874086.34	0.94	0.02
Testis	76220880.69	16629765.78	0.93	0.02
Thyroid	73989411.52	19892076.69	0.88	0.12
Uterus	74771576.02	15422203.59	0.93	0.02
Vagina	69908724.06	15123476.21	0.94	0.02
Whole Blood	80388073.42	22426765.35	0.87	0.12

Supplementary Table 2: Statistics for number and percentage of mapped reads grouped by tissue. Statistics (mean and standard deviation) on the number of mapped reads and ratio between mapped and total number of reads clustered by tissue.

VAR	Pearson.Corr	Pval	AdjR2	VARDESC
DTHVNT	-0.717912412	8.72E-85	0.51447694	Donor On A Ventilator Immediately Prior To Death
MHABNWBC	-0.46167649	9.32E-30	0.21167717	Abnormal Wbc
DTHCERT	-0.408748402	1.76E-22	0.16547655	Death Certificate Available
DTHWTNS	-0.187911449	1.38E-05	0.0334767	Witnessed Death
DTHPLCE	-0.187187721	1.13E-05	0.03325558	Place Of Death
MHOPNWND	-0.173329449	5.22E-05	0.02823685	Open Wounds
DTHLUCODD	-0.169904915	0.02815286	0.02298203	Interval Of Onset To Death For Last Underlying Cause
TRAMP	-0.153917113	0.00031841	0.02188584	Amputation
MHPSBLDCLT	-0.11748701	0.00646711	0.01195639	Positive Blood Cultures
MHNRTHEUR	-0.109517952	0.01140311	0.01013353	Resided On Northern European Military Base
MHDTND72H	-0.101648917	0.01913493	0.00846168	In Detention Center 72h
LBEBVGAB	-0.092119646	0.15482678	0.00432	EBV IgG Ab
MHNPHYS4W	-0.087497227	0.04288145	0.00579744	No Physical Activity 4 Weeks
MHSTD	-0.085777006	0.04757103	0.00549183	Sexually Transmitted Diseases
MHCOCAINE5	-0.084811479	0.05035274	0.00532329	Cocaine Use In 5y
MHNGHTSWT	-0.079752041	0.06528634	0.00449615	Night Sweats
MHLUPUS	-0.07897904	0.06794524	0.00437322	Systemic Lupus
GENDER	-0.07754202	0.07100159	0.00417545	Gender
MHTTOONP	-0.077447675	0.07456451	0.00411912	Non Professional Tattoos
MHOSTMYLTS	-0.074754628	0.08350636	0.00372954	Osteomyelitis
MHPLLABS	-0.074716106	0.08512571	0.00370624	Prescription Pill Abuse
MHHEROIN	-0.067463096	0.11910242	0.00268364	Heroin Use
MHSRGHM	-0.064893267	0.13532875	0.00232874	Resident Of State Run Group Home
MHWTLSUB	-0.063816326	0.14044759	0.00220399	Unexplained Weight Loss
MHSDRGABS	-0.063694585	0.14233307	0.00217786	Signs Of Drug Abuse
MHSKNSPT	-0.060399709	0.16299909	0.0017788	Spots On Skin
MHSCLRDRM	-0.056712063	0.19111831	0.00133908	Scleroderma
MHSUBABSB	-0.047319226	0.27504299	0.00036362	Drugs For Non Medical Use In 5y
MHSXMDB	-0.047294923	0.27483873	0.00036483	Sex For Money Or Drugs
MHSUBABSA	-0.047227872	0.27597182	0.00035497	Drugs For Non Medical Use In 5y
MHIVDRG5	-0.047208229	0.2757205	0.00035663	Intravenous Drug Abuse In 5y
MHCOUGHU	-0.044724985	0.30179627	0.0001279	Unexplained Cough
MHMSXWMA	-0.043229064	0.31827014	-3.91E-06	Men Sex With Men
MHMSXWMB	-0.043229064	0.31827014	-3.91E-06	Men Sex With Men
MHOPPINF	-0.042977428	0.32109794	-2.56E-05	Opportunistic Infections
MHFLU	-0.042438846	0.3262984	-6.47E-05	Influenza (acute viral infection including avian influenza)
MHSMLPXVC	-0.041743857	0.33519637	-0.0001304	Recent Smallpox Vac
DTHATPSY	-0.041091459	0.46245964	-0.0014312	Autopsy Performed By Coroner Or ME
MHBCTINF	-0.039715502	0.35877814	-0.0002924	Bacterial Infections (including septicemia (bacteria in the blood
MHTBHX	-0.03759375	0.38549347	-0.0004602	TB History
MHBLDDND	-0.036134431	0.40377842	-0.0005645	Past Blood Donations Denied
MHTEMPU	-0.032824817	0.44864942	-0.0007967	Unexplained Temperature
MHCANCERC	-0.03233246	0.45633515	-0.0008359	Current Diagnosis Of Cancer
MHEURO5	-0.026845715	0.5359029	-0.0011577	In Europe 5y Since 1980
MHTTOO12M	-0.026715377	0.5378853	-0.0011646	Tattoos Done In 12m
MHSCHZ	-0.022131006	0.60751325	-0.0013646	Schizophrenia
MHPRCNP	-0.022025302	0.61223699	-0.0014008	Non Professional Piercing
MHCLLULTS	-0.020963829	0.6275524	-0.0014254	Cellulites
MHUREMIA	-0.011701795	0.78674015	-0.001732	Uremia (Kidney Disorder)
BMI	-0.010099558	0.81435838	-0.0017462	BMI
MHFNGINF	-0.010095623	0.81527416	-0.0017636	Fungal Infections
MHSZRSU	-0.009575065	0.82528602	-0.0017878	Unexplained Seizures
ETHNCTY	-0.009197384	0.86581993	-0.0028737	Ethnicity
MHRA	-0.007547648	0.86185908	-0.0018226	Rheumatoid Arthritis
MHSRCDSS	-0.007149399	0.86895862	-0.001825	Sarcoidosis
MHASTHMA	-0.00350902	0.93504053	-0.0018395	Asthma
MHMS	-0.002868162	0.94722994	-0.0018679	Multiple Sclerosis

MHUK8096	0.001089381	0.97994447	-0.001875	In Uk 3m 1980 1996
MHSEPSIS	0.002204593	0.95931255	-0.0018608	Documented Sepsis
MHHEPCCT	0.002388046	0.95613611	-0.0018775	Hepatitis C
MHTXCEXP	0.010961789	0.80066545	-0.0017629	Exposure To Toxics
LBCMVTAB	0.012590165	0.83988534	-0.0037168	CMV Total Ab
LBHBSAB	0.015544998	0.94120934	-0.0432261	HBsAb
MHALZDMT	0.019031861	0.6587165	-0.0014924	Alzheimer's OR Dementia
MHCLRD	0.020873936	0.62934955	-0.0014326	Chronic Lower Respiratory Disease
MHFVRU	0.026783998	0.53493194	-0.0011435	High Unexplained Fever
LBHIVO	0.028605797	0.51921455	-0.0011486	HIV I II Plus O Antibody
LBHIVAB	0.029253513	0.51188701	-0.0011306	HIV I II Ab
MHDPRSSN	0.031206354	0.4696845	-0.0008865	Major depression (unipolar depression, major depressive disor
MHARTHTS	0.033751134	0.43294277	-0.0007106	Arthritis
WGHT	0.037950381	0.37744512	-0.0004055	Weight
MHDMNTIA	0.039482283	0.36205976	-0.0003144	Dementia With Unknown Cause
MHHRTDIS	0.040098668	0.35596729	-0.0002759	Ischemic Heart Disease (coronary artery disease (CAD), corona
MHHEPBCT	0.041016527	0.34414604	-0.0001942	Hepatitis B
MHALZHMR	0.044258625	0.30732484	8.28E-05	Alzheimer's
MHPRKNSN	0.045585704	0.28987574	0.00022662	Parkinson's Disease
MHASCITES	0.05060798	0.24125162	0.00070027	Ascites
MHCANCER5	0.055876018	0.19733232	0.0012483	Cancer Diagnosis 5y
MHINFLNE	0.05752017	0.18280421	0.00144907	Infected Lines
MHALS	0.057604309	0.1833931	0.00144831	Amyotropic Lateral Sclerosis
DTHCODD	0.057751947	0.27715326	0.00051985	Interval Of Onset To Death For Immediate Cause
LBEBVMAB	0.065445808	0.31163608	0.00011697	EBV IgM Ab
LBHIV1NT	0.065716966	0.14801157	0.00226153	HIV 1 NAT
MHNEPH	0.06868681	0.11186493	0.00285754	Nephritis, Nephrotic Syndrome and/or Nephrosis
MHT2D	0.06890106	0.1094226	0.00290088	Diabetes mellitus type II (NIDDM, adult onset diabetes)
MHWKNSSU	0.068965152	0.11108505	0.00288894	Unexplained Weakness
HGHT	0.075279171	0.07966598	0.003829	Height
INCEXC	0.078967479	0.06595044	0.00439896	Eligible For Study
TRCRTMPU	0.079485336	0.06419015	0.00448117	Core Body Temperature - Units of measurement
MHCOPD	0.081926764	0.05779144	0.00485538	Chronic Respiratory Disease (Chronic Obstructive Pulmonary Sy
MHWTLSUA	0.091605329	0.03431529	0.00652761	Unexplained Weight Loss
MHCVD	0.095726502	0.02639867	0.00731499	Cerebrovascular Disease (stroke, TIA, embolism, aneurysm, oth
MHSTRDLT	0.098151765	0.02331102	0.00777218	Long Term Steroid Use
LBHBCABM	0.098892412	0.16788835	0.00467548	HBcAb IgM
MHHMPHLIA	0.102789436	0.01739418	0.00870932	Hemophilia
MHBLDOCNT	0.103237853	0.01722074	0.00879137	Received Blood Transfusion In Another Country
MHLVRDIS	0.1075286	0.01249404	0.00972173	Liver Disease (liver abscess, failure, fatty liver syndrome, inheri
MHT1D	0.109376705	0.01097701	0.01012676	Diabetes mellitus type 1 (IDDM, formerly juvenile diabetes)
MHCANCERNM	0.110504942	0.01003551	0.0103821	History Of Non Metastatic Cancer
LBHCV1NT	0.115716892	0.01076003	0.01134773	HCV 1 NAT
DTHFUCODD	0.115972356	0.03103012	0.01058171	Interval Of Onset To Death For First Underlying Cause
TRTPTREF	0.120178369	0.00504485	0.01262111	Tissue Recovery Time Point Reference
MHORGNTP	0.129099097	0.00277506	0.01482167	Received Tissue Organ Transplant
MHHTN	0.132122707	0.00211369	0.01562672	Hypertension
RACE	0.135307172	0.00160827	0.01648671	Race
MHDLYSIS	0.139693259	0.00119759	0.01767465	Dialysis Treatment
DTHVNTD	0.142219562	0.01367928	0.01693857	Time Interval On Ventilator
LBHBCABT	0.153140937	0.00059783	0.02148726	HBcAb Total
LBHBHCVAB	0.160009222	0.00028164	0.02368862	HCV Ab
MHHRTDISB	0.163016025	0.00015683	0.02474103	Heart Disease
DTHCODDU	0.167832159	8.50E-05	0.02637127	Interval Of Onset To Death For Immediate Cause Unit
DTHFUCODDU	0.167832159	8.50E-05	0.02637127	Interval Of Onset To Death For First Underlying Cause Unit
DTHLUCODDU	0.167832159	8.50E-05	0.02637127	Interval Of Onset To Death For Last Underlying Cause Unit
DTHRFGDU	0.167832159	8.50E-05	0.02637127	Number Of Hours In Refrigeration Unit
DTHVNTDU	0.167832159	8.50E-05	0.02637127	Time Interval On Ventilator Unit
MHPNMNIA	0.176176188	4.11E-05	0.02922351	Pneumonia (acute respiratory infection affecting the lungs)
MHPNMIAB	0.179297557	3.13E-05	0.03032492	Pneumonia
MHHRTATT	0.215073981	5.37E-07	0.04446069	Heart attack, acute myocardial infarction, acute coronary syndr
DTHDTRMN	0.218812589	2.61E-07	0.04611902	Person Who Determined Date Time Of Death
MHRNLFLR	0.221482519	2.16E-07	0.04727704	Renal Failure
DTHMNNR	0.224077434	1.31E-07	0.04845508	Manner Of Death
DTHTPTREF	0.249824598	3.59E-09	0.06067927	Death Time Point Reference
TRCRTMP	0.29637168	1.51E-08	0.08522252	Core Body Temperature
AGE	0.301761477	6.79E-13	0.08937988	Age
MHSRC	0.32205546	1.42E-14	0.10206301	Primary History Source
DTHRFGD	0.578168034	0.00034196	0.31347447	Number Of Hours In Refrigeration
COHORT	0.619157175	0	0.38221578	Cohort
DTHHRDY	0.631692863	0	0.39788899	Hardy Scale
TRVNTSR	0.686413731	0	0.47018629	On ventilator less than 24 hours
DTHRFG	0.73650546	0	0.54068719	Body Refrigerated
TRCCLMPD	0.932937701	0	0.86979918	Time Cross Clamp Applied (Minutes)
TRCHSTIND	0.976864896	0	0.95417808	Time of Chest Incision (Minutes)
TRDNISCH	0.989196047	0	0.9784691	Total Ischemic time for a donor

Supplementary Table 3: Correlation values between different covariates and PMI values. Correlation of the different covariates annotated for each sample and respective individual and the Post-mortem interval (in minutes).

Tissue	RIN_PMI_Co
Esophagus - Mucosa	-0.6552697
Liver	-0.6017417
Esophagus - Muscularis	-0.5917906
Colon - Transverse	-0.5689093
Colon - Sigmoid	-0.5678484
Ovary	-0.5425091
Heart - Atrial Appendage	-0.5419786
Heart - Left Ventricle	-0.5123034
Uterus	-0.511845
Vagina	-0.5070686
Lung	-0.503298
Prostate	-0.4715444
Esophagus - Gastroesophageal Jun	-0.4663229
Artery - Coronary	-0.450632
Adipose - Visceral (Omentum)	-0.4480063
Kidney - Cortex	-0.3962778
Adrenal Gland	-0.3873137
Brain - Cortex	-0.38653
Breast - Mammary Tissue	-0.3854187
Testis	-0.384105
Minor Salivary Gland	-0.3794755
Thyroid	-0.2898137
Adipose - Subcutaneous	-0.2804226
Artery - Aorta	-0.2650855
Brain - Cerebellum	-0.2614623
Pancreas	-0.2318895
Artery - Tibial	-0.203151
Whole Blood	-0.1978432
Muscle - Skeletal	-0.1964504
Stomach	-0.1890257
Small Intestine - Terminal Ileum	-0.1423032
Nerve - Tibial	-0.099228
Spleen	-0.0894587
Skin - Not Sun Exposed (Suprapubi	-0.0290026
Pituitary	-0.0141668
Skin - Sun Exposed (Lower leg)	0.04114183

Supplementary Table 4: Correlation of RIN with PMI per Tissue.

Covariate	Description
AGE	Age
HGHT	Height
WGHT	Weight
BMI	Body Mass Index
ETHNCTY	Ethnicity
GENDER	Gender
MHCANCERNM	History Of Non Metastatic Cancer
SMRIN	RIN Number
SMTASSCR	Autolysis Score
SMCAT	Category or classifier of a set of responses, indicates the color of the sample collection kit
SMCENTER	Code for BSS collection site
SMTSTPTREF	Time point reference for Start and End times of sample procurement
SMNABTCH	Nucleic Acid Isolation Batch ID
COHORT	Cohort

Supplementary Table 5: Set of selected covariates (Pearson correlation with PMI > 0.1) used for regression analysis of PMI and gene expression.

PMI correlation greater than 0.2 or 0.3; Analysis with included covariates									Genes with PMI correlation without covars			
**Tissue**	**Total**	**|r|>0.2**	**%|r|>0.2**	**|r|>0.3**	**min-r**	**max-r**	**iqr**	**FDR < 1%**	**|r|>0.2**	**% of |r|>0.2**	**FDR < 1%**	**% FDR < 1%**
Adipose - Subcutaneous	17283	368	2.13	6	-0.30	0.37	0.12	475	6575	38.04	8875	51.35
Adipose - Visceral (Omentum)	17393	21	0.12	0	-0.23	0.22	0.09	0	9678	55.64	10383	59.7
Adrenal Gland	17111	73	0.43	0	-0.27	0.27	0.10	0	6777	39.61	4757	27.8
Artery - Aorta	16631	24	0.14	0	-0.26	0.22	0.10	0	8710	52.37	9264	55.7
Artery - Coronary	17208	164	0.95	0	-0.29	0.27	0.12	0	8934	51.92	7247	42.11
Artery - Tibial	16078	99	0.62	1	-0.25	0.38	0.10	1	3336	20.75	5022	31.24
Brain - Cerebellum	18346	149	0.81	3	-0.30	0.31	0.10	0	225	1.23	0	0
Brain - Cortex	17867	954	5.34	5	-0.31	0.33	0.17	0	888	4.97	0	0
Breast - Mammary Tissue	18397	6	0.03	0	-0.21	0.20	0.10	0	7011	38.11	7163	38.94
Colon - Sigmoid	17185	16	0.09	0	-0.23	0.23	0.09	0	10067	58.58	9198	53.52
Colon - Transverse	18300	0	0.00	0	-0.14	0.14	0.07	0	11093	60.62	11281	61.64
Esophagus - Gastroeso.	16997	13	0.08	0	-0.23	0.22	0.09	0	9577	56.35	8705	51.21
Esophagus - Mucosa	16990	0	0.00	0	-0.18	0.16	0.07	0	9472	55.75	10880	64.04
Esophagus - Muscularis	16763	0	0.00	0	-0.20	0.17	0.07	0	9588	57.2	10571	63.06
Heart - Atrial Appendage	16301	20	0.12	0	-0.22	0.24	0.10	0	8762	53.75	8791	53.93
Heart - Left Ventricle	15167	16	0.11	0	-0.22	0.24	0.09	0	10071	66.4	10501	69.24
Kidney - Cortex	18189	866	4.76	19	-0.33	0.34	0.15	0	10512	57.79	966	5.31
Liver	15409	195	1.27	0	-0.28	0.29	0.13	0	9736	63.18	8249	53.53
Lung	18716	9	0.05	0	-0.22	0.22	0.10	0	11210	59.9	12967	69.28
Minor Salivary Gland	18294	179	0.98	0	-0.28	0.28	0.12	0	7085	38.73	77	0.42
Muscle - Skeletal	14106	315	2.23	0	-0.29	0.29	0.14	1277	5936	42.08	8580	60.83
Nerve - Tibial	18258	85	0.47	0	-0.26	0.26	0.10	0	6076	33.28	7915	43.35
Ovary	17840	40	0.22	0	-0.24	0.25	0.09	0	9825	55.07	6691	37.51
Pancreas	15921	4	0.03	0	-0.24	0.19	0.08	0	1280	8.04	150	0.94
Pituitary	19568	1847	9.44	79	-0.39	0.38	0.17	0	3025	15.46	0	0
Prostate	19296	2	0.01	0	-0.19	0.20	0.08	0	7173	37.17	3217	16.67
Skin - Not Sun Exposed	17810	5	0.03	0	-0.20	0.24	0.06	0	7192	40.38	8099	45.47
Skin - Sun Exposed (Lower leg)	17777	37	0.21	2	-0.28	0.35	0.09	4	3940	22.16	6146	34.57
Small Intestine - Terminal Ileum	18904	69	0.37	0	-0.27	0.28	0.08	0	2137	11.3	0	0
Spleen	18323	80	0.44	0	-0.29	0.29	0.10	0	422	2.3	0	0
Stomach	17803	237	1.33	0	-0.27	0.27	0.16	0	4724	26.53	3749	21.06
Testis	25331	80	0.32	0	-0.24	0.24	0.16	0	12636	49.88	11671	46.07
Thyroid	18902	30	0.16	0	-0.25	0.23	0.09	0	7580	40.1	9771	51.69
Uterus	17907	57	0.32	0	-0.26	0.24	0.10	0	10030	56.01	5763	32.18
Vagina	18522	58	0.31	1	-0.28	0.31	0.11	0	6920	37.36	2598	14.03
Whole Blood	13486	697	5.17	61	-0.35	0.48	0.13	192	907	6.73	412	3.06
**Average**	**17621.64**	**189.31**	1.07	**4.92**				**54.14**	**6919.72**	**39.30**	**6101.64**	**34.98**
**Sum**		**6815**		**177**				**1949**				

Supplementary Table 6: Correlation values of gene expression and PMI per tissue. Correlation values of gene expression and pmi per tissue. Both models with and without covariates are considered. Number of genes tested per tissues, (|r|>0.2) number of genes with |r|>0.2, (|r|>0.3) number of genes with |r|>0.3, (min-r) minimum value of r observed among all genes, (max-r) maximum value of r observed among all genes, (iqr) interquartile range for all correlation values, number of genes where only an FDR < 1%is considered as a filter. % of |r|>0.2, percentage of genes with |r|>0.2; % FDR < 1%, percentage of genes where only an FDR < 1%is considered as a filter.

Tissue	NumExons	GC_content	length_CDS	length_Gene
Kidney - Cortex	-0.06	-0.26	-0.09	0.04
Minor Salivary Gland	-0.06	0.05	-0.05	0.01
Uterus	-0.1	0.31	-0.15	-0.11
Vagina	-0.04	0.02	-0.02	-0.05
Small Intestine - Terminal Ileum	-0.06	0.03	-0.09	0
Ovary	0.04	-0.05	0	0.08
Spleen	0.03	0.1	0.01	-0.02
Prostate	0.02	-0.02	0	0.04
Pituitary	0.02	-0.07	-0.02	0.05
Liver	-0.09	0.05	-0.08	0
Artery - Coronary	0.07	-0.11	0.09	0.11
Adrenal Gland	0.02	0.1	0	0.01
Colon - Sigmoid	-0.04	0.09	-0.05	0.05
Esophagus - Gastroesop.	0.02	0.09	0.02	0.07
Pancreas	0.03	-0.14	0	0.09
Testis	-0.09	-0.06	-0.19	-0.01
Colon - Transverse	0.09	-0.01	0.13	0.11
Stomach	0.12	-0.03	0.15	0.17
Breast - Mammary Tissue	0.05	0.04	0.06	0.04
Heart - Atrial Appendage	-0.03	0.01	-0.02	0.06
Adipose - Visceral (Omentum)	-0.04	0.08	-0.06	0.03
Artery - Aorta	0.01	-0.05	0.04	0.11
Skin - Not Sun Exposed	-0.03	-0.27	-0.04	0.07
Esophagus - Muscularis	0.01	0.08	-0.04	0.04
Heart - Left Ventricle	-0.08	0.07	-0.09	-0.04
Esophagus - Mucosa	0.03	0.21	0.05	0
Nerve - Tibial	0.06	-0.05	0.1	0.13
Adipose - Subcutaneous	0.1	-0.08	0.08	0.11
Thyroid	-0.03	0.08	-0.11	0.05
Artery - Tibial	0.04	-0.06	0.03	0.13
Skin - Sun Exposed (Lower leg)	0.08	-0.15	0.05	0.08
Lung	0.1	-0.06	0.12	0.17
Muscle - Skeletal	-0.01	-0.03	-0.02	0.07
Whole Blood	0.08	0.08	0.09	0.06
Brain - Cortex	0.02	-0.18	0.01	0.08
Brain - Cerebellum	-0.04	-0.02	0	0

Supplementary Table 7: Correlation values with different gene features of r (correlation of PMI and gene expression residuals).

**Fold-Change > 2**				
**PostMortem Samples (PMI interval)**	**Up in Pre-mortem**	**Up in Pre-mortem (Pcoding)**	**Up in Post-mortem**	**Up in Post-mortem (Pcoding)**
All	964	759	1377	1173
>0 & <=406	547	430	1039	911
>406 & <=635	1907	1581	1940	1646
>635 & <=867	1767	1471	1973	1693
>867 & <=1401	244	185	933	795

**Fold-Change > 3**				
**PostMortem Samples (PMI interval)**	**Up in Pre-mortem**	**Up in Pre-mortem (Pcoding)**	**Up in Post-mortem**	**Up in Post-mortem (Pcoding)**
All	223	154	583	506
>0 & <=406	109	77	416	368
>406 & <=635	828	635	885	741
>635 & <=867	751	577	942	802
>867 & <=1401	11	8	424	366

Supplementary Table 8: Differential expression in Pre versus Post mortem blood samples. Number of differentially expressed genes for the comparison of pre and post-mortem samples, where the post-mortem samples are divided in four different intervals (equivalent size) and compares separately with the pre-mortem samples. Values considering all samples in the two groups are also presented.

Path/Term	UP/DOWN	Statistic	p-value	FDR p-value
Blood coagulation	UP	15,81	2,60E-56	1,52E-54
Hemostasis	UP	15,81	2,60E-56	1,52E-54
Fibrinolysis	UP	15,00	6,92E-51	1,01E-49
Plasminogen activation	UP	14,49	1,26E-47	1,05E-46
Collagen degradation	UP	11,29	1,45E-29	4,34E-29
Necrosis	UP	7,00	2,49E-12	4,48E-12
Biological rhythms	DOWN	-12,36	3,90E-35	1,76E-34
Jnfolded protein response	UP	12,41	2,16E-35	1,05E-34
ER-Golgi transport	UP	11,83	2,71E-32	1,02E-31
DNA damage	UP	11,73	8,48E-32	3,01E-31
DNA repair	UP	7,70	1,29E-14	2,55E-14
Stress response	DOWN	-11,15	6,63E-29	1,94E-28
Pyrogen	DOWN	-8,23	1,83E-16	3,82E-16
Adaptive immunity	DOWN	-11,47	1,76E-30	5,55E-30
Inflammatory response	DOWN	-11,93	7,82E-33	3,05E-32
Antiviral defense	DOWN	-12,03	2,28E-33	9,51E-33
Innate immunity	DOWN	-14,00	1,44E-44	8,86E-44
Immunity	DOWN	-14,01	1,27E-44	8,25E-44
Prostaglandin biosynthesis	UP	14,63	1,74E-48	1,70E-47
Prostaglandin metabolism	UP	14,63	1,74E-48	1,70E-47
Fatty acid biosynthesis	UP	14,20	8,56E-46	6,26E-45
Glycogen biosynthesis	UP	11,80	3,90E-32	1,43E-31
Lipid biosynthesis	UP	11,55	7,17E-31	2,33E-30
Glycolysis	UP	10,91	9,33E-28	2,54E-27
Fatty acid metabolism	UP	10,89	1,25E-27	3,34E-27
Lipid degradation	UP	10,49	9,03E-26	2,25E-25
Lipid metabolism	UP	10,32	5,46E-25	1,33E-24
Tricarboxylic acid cycle	DOWN	-12,14	6,34E-34	2,75E-33
Glycogen metabolism	DOWN	-14,60	2,52E-48	2,26E-47
Carbohydrate metabolism	DOWN	-15,02	5,14E-51	8,58E-50
Protein biosynthesis	UP	7,20	5,73E-13	1,05E-12
cAMP biosynthesis	DOWN	-7,36	1,81E-13	3,42E-13
Cholesterol biosynthesis	DOWN	-9,39	5,52E-21	1,24E-20
Sterol biosynthesis	DOWN	-9,39	5,52E-21	1,24E-20
Steroid biosynthesis	DOWN	-9,39	5,52E-21	1,24E-20
Neurogenesis	DOWN	-9,57	1,01E-21	2,42E-21
eurotransmitter biosynthe	DOWN	-14,89	3,48E-50	4,07E-49
Initiation factor	DOWN	-15,72	1,03E-55	4,02E-54
alcium channel (inhibition	UP	11,64	2,46E-31	8,48E-31
alcium transport (inhibitio	UP	11,40	4,06E-30	1,25E-29
Transport (inhibition)	DOWN	-7,27	3,56E-13	6,62E-13
Potassium channel	DOWN	-7,75	8,92E-15	1,80E-14
Sodium channel	DOWN	-10,59	3,28E-26	8,35E-26
Antiport	DOWN	-10,99	4,02E-28	1,12E-27
Ion channel	DOWN	-12,79	1,75E-37	9,33E-37
Ion transport	DOWN	-13,14	1,85E-39	1,08E-38
Symport	DOWN	-14,89	3,48E-50	4,07E-49
Voltage-gated channel	DOWN	-15,25	1,50E-52	3,51E-51
Potassium transport	DOWN	-15,51	2,66E-54	7,78E-53
Initiation factor	DOWN	-15,72	1,03E-55	4,02E-54
Cell cycle (inhibition)	UP	15,02	5,07E-51	8,58E-50
Growth arrest	UP	13,05	5,75E-39	3,21E-38
Meiosis (inhibition)	UP	12,77	2,31E-37	1,18E-36
Chromosome partition	UP	12,37	3,53E-35	1,65E-34
DNA replication (inhibition	UP	8,88	6,66E-19	1,47E-18
Angiogenesis (inhibition)	UP	8,72	2,57E-18	5,47E-18
RNA processing (inhibitio	UP	8,03	9,53E-16	1,96E-15
Chondrogenesis	UP	7,69	1,41E-14	2,76E-14
Protein biosynthesis	UP	7,20	5,73E-13	1,05E-12

Supplementary Table 9: Functional processes differentially activated in post-mortem blood. Functional processes differentially activated in post-mortem Blood. Terms obtained with the HiPathia program include term or pathway, status of activation (up or down) in the post-mortem samples, statistic score, p-value and FDR.

tissue	totalgenes	significantGenes	%SignifGenes
Artery - Tibial	14251	0	0
Brain - Cerebellum	15392	0	0
Brain - Cortex	15761	0	0
Muscle - Skeletal	13578	0	0
Nerve - Tibial	15407	0	0
Pancreas	14815	0	0
Pituitary	16151	0	0
Spleen	15479	0	0
Stomach	14807	0	0
Adipose - Subcutaneous	14829	1	0
Minor Salivary Gland	15555	5	0
Skin - Sun Exposed (Lower leg)	15268	5	0
Small Intestine - Terminal Ileum	15763	6	0
Adrenal Gland	15059	8	0.1
Testis	19073	18	0.1
Thyroid	15481	21	0.1
Colon - Transverse	15216	30	0.2
Prostate	15637	58	0.4
Vagina	15156	77	0.5
Skin - Not Sun Exposed	15246	78	0.5
Breast - Mammary Tissue	15036	81	0.5
Kidney - Cortex	15472	130	0.8
Lung	15396	195	1.3
Adipose - Visceral (Omentum)	14958	221	1.5
Heart - Atrial Appendage	14805	268	1.8
Esophagus - Gastroesop.	14917	290	1.9
Artery - Aorta	14690	300	2
Esophagus - Muscularis	14940	331	2.2
Uterus	15125	412	2.7
Artery - Coronary	14877	484	3.3
Whole Blood	12194	557	4.6
Ovary	14964	586	3.9
Colon - Sigmoid	15026	678	4.5
Heart - Left Ventricle	14100	702	5
Esophagus - Mucosa	15105	1057	7
Liver	14184	1534	10.8
**Average**			**1.5**

Supplementary Table 10: Number of genes with significant correlation of splicing entropy with PMI per tissue. Tables present the number of tested genes, number of significant genes and percentage of significant genes over the total genes.

Prefix	Description
SMNTRART	Intragenic Rate: The fraction of reads that map within genes (within introns or exons)
SMEXNCRT	Exonic Rate: The fraction of reads that map within exons
SMNTERRT	Intergenic Rate: The fraction of reads that map to the genomic space between genes
SMNTRNRT	Intronic Rate: The fraction of reads that map within introns
SMEXPEFF	Expression Profiling Efficiency: Ratio of exon reads to total reads
SMMAPRT	Mapping Rate: Ratio of total mapped reads to total reads
SMRRNART	rRNA Rate: Ratio of all reads aligned to rRNA regions to total reads
SM550NRM	5' 50-based normalization: 50 (this number is the value for the transcript end length parameter) refers to the definition of how many bases are considered at the end; this value is the ratio between the coverage at the 5' end and the average coverage of the full transcript, averaged over all transcripts;
SM350NRM	3' 50-base normalization: the ratio between the coverage at the 3' end and the average coverage of the full transcript, averaged over all transcripts

Supplementary Table 11: RNA-seq mapping metrics derived from RNA-SeQC.

Supplementary references

1 Benjamini, Y. & Hochberg, Y. Controlling the false discovery rate: a practical and powerful approach to multiple testing. *Journal of the Royal Statistical Society Series B* **57**, 289–300, (1995).

2 Hidalgo, M. R. *et al*. High throughput estimation of functional cell activities reveals disease mechanisms and predicts relevant clinical outcomes. *Oncotarget* **8**, 5160-5178, (2017).
