# Supplementary material for: The effects of death and post-mortem cold ischemia on human tissue transcriptomes
Source: Nat Commun. 2018 Feb 13;9:490. doi: 10.1038/s41467-017-02772-x (PMC5811508; doi:10.1038/s41467-017-02772-x)
Supplement: Supplementary file 3 — Description of Additional Supplementary Files [file 41467_2017_2772_MOESM3_ESM.pdf]

Description of Supplementary Files

File Name: Supplementary Data 1
Description: List of the genes in the different tissues with significant temporal change across the different tissues.

File Name: Supplementary Data 2
Description: Differential expression genes for the comparison of pre and post-mortem samples (considering all samples in the two groups).
